# Inhibitor of Kappa B Epsilon (IκBε) Is a Non-Redundant Regulator of c-Rel-Dependent Gene Expression in Murine T and B Cells

**DOI:** 10.1371/journal.pone.0024504

**Published:** 2011-09-06

**Authors:** Joanna M. Clark, Karolina Aleksiyadis, Alex Martin, Kay McNamee, Tharsana Tharmalingam, Richard O. Williams, Sylvie Mémet, Andrew P. Cope

**Affiliations:** 1 Academic Department of Rheumatology, Centre for Molecular and Cellular Biology of Inflammation, Division of Immunology, Infection and Inflammatory Diseases, School of Medicine, King's College London, University of London, London, United Kingdom; 2 The Kennedy Institute of Rheumatology Division, Imperial College London, London, United Kingdom; 3 Institut Pasteur, Unité de Mycologie Moléculaire, Paris, France; 4 Centre National de la Recherche Scientifique, Unité de Recherche Associée 3012, Paris, France; Chinese University of Hong Kong, Hong Kong

## Abstract

Inhibitors of kappa B (IκBs) -α, -β and -ε effect selective regulation of specific nuclear factor of kappa B (NF-κB) dimers according to cell lineage, differentiation state or stimulus, in a manner that is not yet precisely defined. Lymphocyte antigen receptor ligation leads to degradation of all three IκBs but activation only of subsets of NF-κB-dependent genes, including those regulated by c-Rel, such as anti-apoptotic CD40 and BAFF-R on B cells, and interleukin-2 (IL-2) in T cells. We report that pre-culture of a mouse T cell line with tumour necrosis factor-α (TNF) inhibits IL-2 gene expression at the level of transcription through suppressive effects on NF-κB, AP-1 and NFAT transcription factor expression and function. Selective upregulation of IκBε and suppressed nuclear translocation of c-Rel were very marked in TNF-treated, compared to control cells, whether activated via T cell receptor (TCR) pathway or TNF receptor. IκBε associated with newly synthesised c-Rel in activated cells and, in contrast to IκBα and -β, showed enhanced association with p65/c-Rel in TNF-treated cells relative to controls. Studies in IκBε-deficient mice revealed that basal nuclear expression and nuclear translocation of c-Rel at early time-points of receptor ligation were higher in IκBε−/− T and B cells, compared to wild-type. IκBε−/− mice exhibited increased lymph node cellularity and enhanced basal thymidine incorporation by lymphoid cells *ex vivo*. IκBε−/− T cell blasts were primed for IL-2 expression, relative to wild-type. IκBε−/− splenic B cells showed enhanced survival *ex vivo*, compared to wild-type, and survival correlated with basal expression of CD40 and induced expression of CD40 and BAFF-R. Enhanced basal nuclear translocation of c-Rel, and upregulation of BAFF-R and CD40 occurred despite increased IκBα expression in IκBε−/− B cells. The data imply that regulation of these c-Rel-dependent lymphoid responses is a non-redundant function of IκBε.

## Introduction

Members of the nuclear factor of kappa B (NF-κB) transcription factor family, p50, p52, p65 (RelA), c-Rel and RelB, are maintained as inactive homo- or heterodimers in the cytoplasm by inhibitors of kappa B (IκBs). Interactions are via ankyrin repeats of IκB and Rel homology domains (RHD) of NF-κB [1], [2]. The RHDs contain dimerisation, nuclear localisation and DNA-binding regions, and c-Rel, p65 and RelB, but not p50 and p52, also have transactivation domains which confer transcriptional activity. c-Rel, p65 and p50 are regulated by IκBs -α, -β and -ε whose phosphorylation, ubiquitination and degradation are effected following activation of the IκB kinase (IKK) complex, particularly IKK2, when the classical NF-κB pathway is activated [3]. c-Rel, p65 and p50 also associate with the intramolecular IκB domains of p105 and p100, precursor proteins of p50 and p52 respectively. p50 is generated through constitutive processing, and p52 upon activation of the alternative, IKK1-dependent pathway, which also involves RelB. IκB degradation permits nuclear translocation of NF-κB and induction of genes responsible for many cellular responses including inflammation, survival and differentiation [4], [5], [6].

Activated NF-κB itself induces resynthesis of all three IκBs. How, then, are signal-, cell- and subunit-specific regulation of NF-κB dimers and their target genes brought about? Studies suggest that the necessary specificity may be achieved via differential expression - in space and time - of IκB isoforms. Thus, the rate of degradation and resynthesis of each isoform may vary with stimulus and cell-type [7], and according to the differentiation status of a cell. IκBε, for example, is upregulated in HL60 cells as they undergo terminal differentiation and IκBε is expressed at high levels in both naïve murine splenic B cells and IgM+ mouse B cell lines, relative to IgG+ cells [8], [9]. IκBε differs from IκBs -α and -β structurally [8]; and IκBs differ from one another in preferential NF-κB binding and mechanism of action. Thus, IκBα tends to bind p65/p50, but IκBs -ε and -β prefer c-Rel and p65 [8], [10], [11], [12], [13], [14]. Further, although IκBε associates with p50 and p52 *in vivo* and *in vitro*, it is DNA-binding of cRel/p50 and cRel or p65 dimers which it inhibits [15], suggesting that IκBε regulates genes transactivated by heterodimers of c-Rel and homodimers of RelA. Functionally, IκBε and IκBα both contribute to post-induction repression of transcription by removing NF-κB from the nucleus, whereas newly-synthesised IκBβ forms a ternary complex with NF-κB on DNA to maintain its transcriptional activity [16], [17]. Recent studies suggest that differential regulation of IκBε may also be effected through interactions with the protein phosphatase 6 (PP6) ternary complex [18], [19], [20].

c-Rel, the prototype member of the NF-κB family, is expressed in lymphoid, myelomonocytic and erythroid cells in haematopoietic organs of both adult and foetus, and is essential for normal function of B and T cells, macrophages and dendritic cells. Its expression and activation are regulated according to cell lineage, developmental and differentiation state, as well as stimulus received by the cell [21], [22]. For example, c-Rel is constitutively cytosolic in naïve B lymphocytes but nuclear in mature B cells, an altered state attributed to an increased rate of degradation of IκBα in mature B cells [23] but which perhaps better reflects the down-regulation of IκBε in the latter [9]. As a second example, the cytosolic pool of c-Rel in resting T lymphocytes is not translocated instantly to the nucleus following stimulation via the T cell receptor (TCR). Rather, newly synthesised c-Rel only appears in the nucleus after 1.5−2 hours of TCR activation [24]. This study, which predated the discovery of IκBε, suggested that differential subunit- and cell-specific regulation of NF-κB was effected by IκBα and -β. Thus, mechanisms of temporal and spatial regulation by IκB of c-Rel have not been fully defined in lymphocytes.

c-Rel upregulates genes involved in survival, proliferation or differentiation including c-Rel itself, interleukin- 2 (IL-2) in T cells, and genes involved in B cell survival [22], [25], [26]. AP-1 and NFAT transcription factors are also required for full activation of the IL-2 proximal promoter (pIL-2), but c-Rel is essential for IL-2 expression [27], [28], [29], [30]. c-Rel is required for induction of CD40. CD40 was originally detected on B lymphocytes but has since been shown to be expressed on T cells and antigen presenting cells such as dendritic cells, as well as non-haematopoietic endothelial cells and fibroblasts [26], [31], [32], [33]. Ligation of CD40 on B cells delivers anti-apoptotic, maturation and proliferative signals to the B cell [34]. Prolonged activation of c-Rel, such as occurs during B cell maturation, also promotes B cell receptor-dependent induction of B cell activating factor receptor (BAFF-R) and its downstream target p100/NF-κB2, rendering B cells competent for the anti-apoptotic BAFF signal [35], [36]. Furthermore, both CD40 and BAFF-R have been reported to interact and cooperate with nuclear c-Rel, but not p65, to upregulate their own ligands on B cells [37], [38].

An earlier focus of our laboratory has been the study of the effects of tumour necrosis factor-α (TNF), whose expression is dysregulated in inflammatory diseases such as rheumatoid arthritis, on T cell function [39], [40]. Specifically, we wanted to elucidate mechanisms whereby TNF caused profound, reversible inhibition of subsequent IL-2 induction in mouse T cell hybridomas [41], [42]. In the work presented here, we show that induction of IL-2 was suppressed at the level of transcription and that TNF inhibited PMA and ionomycin-induced expression, nuclear translocation and transcriptional activity of AP-1, NFAT and NF-κB to a greater or lesser extent. TNF pre-treatment also inhibited NF-κB signalling when cells were subsequently restimulated with TNF. Thus prolonged culture in TNF affected 11A2 T cell signalling in a number of ways, many of which were likely to contribute to the profound suppression of IL-2 production

In the course of these studies, however, we became most interested in the two clearest and most consistent effects of TNF, which were a very marked inhibition of nuclear translocation of c-Rel, compared to p65, AP-1 or NFAT and, secondly, selective upregulation and impaired degradation of IκBε, out of the IκBs. We wondered to what extent the first phenomenon was a consequence of the second and wanted to explore whether this was an example of selective regulation of c-Rel by IκBε in lymphocytes. Our hypothesis was that, in the absence of IκBε, c-Rel would appear in the nucleus of unstimulated and naïve lymphocytes and the cells would therefore show altered expression of c-Rel-dependent genes. We found increased nuclear expression of c-Rel in unstimulated T cell blasts and in naïve splenic B cells from IκBε-deficient mice, despite increased basal expression of IκBα in these mice. IκBε deficiency was associated with increased lymph node cellularity and basal thymidine incorporation of lymphoid cells *ex vivo*. We observed increased lymph node B cell numbers *in vivo* and enhanced B lymphocyte survival *ex vivo* which was associated with upregulation of CD40 and BAFF-R in IκBε−/− mice. The data suggest that negative regulation of these c-Rel-dependent pro-survival genes is a non-redundant function of IκBε in B cells. We propose that modulation of IκBε expression and degradation is an important mechanism whereby the fine-tuning of appropriate c-Rel activity is achieved in lymphoid cells.

## Materials and Methods

### Antibodies and reagents

Agonistic anti-CD3ε mAb 145-2C11, and rat anti-mouse IL-2 antibody pairs were from BD-Biosciences, Oxford, UK. Antibodies against c-Fos, Fos B, Fra-1, Fra-2, c-Jun, Jun B, Jun D, NFAT2 (NFATc1, clone 7A2), p65/RelA, c-Rel, p50 (NLS), and IκBs -α, -β and -ε, used for immunoblots, EMSA supershift and immunoprecipitation, were all from Santa Cruz Biotechnology (Insight Biotechnology, Wembley, UK). Agarose-conjugated anti-c-Rel and anti-p65 Abs for immunoprecipitation were also from Santa Cruz. Anti-NFAT1 (NFATp, NFATc2) antibody was from Affinity Bioreagents™ (Thermo Fisher Scientific, Loughborough, UK). HRP-linked antibodies for immunoblot were all from DAKO (DAKO UK, Ely, UK). All flow cytometry antibodies were from eBioscience (eBioscience Ltd, Hatfield, UK). EMSA oligonucleotide probes: AP-1 5′-CGCTTGATGAGTCAGCCGGAA-3′; NF-κB 5′-AGTTGAGGGGACTTTCCCAGG-3′ were both from Promega (Promega UK, Southampton, UK); CD28RR 5′-TTTAAAGAAATTCCAAAGAGTCATCA-3′ (forward and reverse primers from MWG (Eurofins MWG Operon, Ebersberg, Germany); NFAT/AP-1 5′-CGCCCAAAGAGGAAAATTTGTTTCATA-3′ (Santa-Cruz/Insight Biotechnology, Wembley, UK). Other reagents were from Sigma-Aldrich Company Ltd (Dorset, UK) or BDH (VWR International, Lutterworth, UK).

### Cells and Cell Culture

The derivation and culture of mouse T cell hybridoma clone 11A2, which is specific for human cartilage glycoprotein-39 (HCgp-39), restricted by HLA-DR4, and expresses human CD4, has been described [43]. 11A2 cells were cultured in RPMI 1640 supplemented with 25 mM HEPES, 2 mM L-glutamine, 10% heat-inactivated foetal calf serum, 100 U/ml penicillin, 100 µg/ml streptomycin, 1 mM sodium pyruvate and 50 µM 2-mercaptoethanol, at 37°C and 5% CO_2_. Cells were passaged every 48 hours into fresh complete medium, in the presence or absence of recombinant mouse TNF 2.5 ng/ml (BD Biosciences). Mouse B cell line A20 and B cell hybridoma SP2/0 were cultured in RPMI containing 10% heat-inactivated foetal calf serum, 100 U/ml penicillin, 100 µg/ml streptomycin and 50 µM 2-mercaptoethanol, in the presence or absence of mTNF 20 ng/ml for 8 days at 37°C and 5% CO_2._

### Cell Stimulation and IL-2 assay

11A2 cells were harvested, washed and resuspended in complete medium at 10^6^ cells/ml in the absence of TNF, prior to incubation with either plate-bound anti-CD3ε, or with PMA and ionomycin (Calbiochem, Merck Biosciences, Beeston, UK) at the concentrations and for the times indicated. IL-2 protein in cell supernatants was assayed by fluorescent immunosorbant assay, as described [41].

### Ribonuclease protection assay and RNA stability

Control and TNF-treated 11A2 cells were resuspended in complete medium at 10^6^ cells/ml in the absence of TNF, then incubated with either plate-bound anti-CD3ε 10 µg/ml, or PMA and ionomycin for the times indicated. Cells were harvested, washed in ice-cold PBS and total RNA extracted (QIAamp® RNA Blood Mini Kit, Qiagen, Crawley, UK). mRNA species were visualised by ribonuclease protection assay (BD RiboQuant™ kit mCK-1, BD Biosciences) and phosphor-imaging (Fuji FLA 2000). For stability studies, actinomycin D 10 µg/ml was added after 4 hours' stimulation, and cells were harvested at the times indicated, washed in ice-cold PBS and snap-frozen until RNA was extracted and analysed.

### Transfections and reporter assays

Control and TNF-treated 11A2 T cells (5×10^6^−10^7^ cells per point) were transfected using Amaxa® Nucleofector® I, Kit V®, programme A10 (Amaxa®, Lonza Wokingham, Ltd, UK) with 10 µg firefly luciferase reporter vectors pAP-1-TA-luc (TRE consensus sequence), pNFAT-TA-luc (ARRE-2 from mouse pIL-2), or pNF-κB-TA-luc (consensus response element of mouse MHCII invariant chain) (all from Takara Bio Europe/Clontech, Lonza Wokingham, Ltd, UK). In all cases, cells were co-transfected with 0.2 µg phRL-SV40 expressing Renilla luciferase (Promega UK), to normalise data for transfection efficiency. **Note:** Among many other transcription factor binding sites, the SV40 promoter/enhancer of phRL-SV40 contains two NF-κB consensus sequences with the potential for interference in NF-κB-dependent promoter assays. However, transfecting with the lowest feasible amount of phRL-SV40 DNA, we found the stimulation index (due to PMA and ionomycin) of renilla luciferase expression was similar for control and TNF-treated cells in all experiments. Thus any differences seen between control and TNF-treated cells in terms of firefly luciferase normalised to renilla luciferase expression were independent of effects of TNF pre-treatment on phRL-SV40. Transfected cells were plated out at 10^6^/ml, allowed to recover for two hours, then stimulated with PMA 50 ng/ml and ionomycin 100 ng/ml for up to 6 hours. Cell lysates were assayed for luminescence due to firefly and renilla luciferase activities (Dual-Luciferase Reporter™ assay, Promega UK).

### Generation of pFLAG-CMV4-c-Rel

The murine c-Rel sequence was digested from cloning vector pSPORT (Invitrogen, Paisley, UK), end-filled to allow cloning into expression vector pFLAG-CMV4 (Sigma-Aldrich) and repaired for a ‘stop’ codon mutation at the end of the rel homology domain before sequencing across pFLAG-c-Rel (Eurofins MWG Operon, London, UK). Expression of c-Rel and HA immunoreactivity of the correct size was confirmed by immunoblot following lipofection of NIH-3T3 fibroblasts. For reconstitution experiments in 11A2 cells, 2 µg of pFLAG-CMV4-c-Rel or empty vector were used.

### EMSAs, immunoblots, and immunoprecipitations

Control and TNF-treated cells (10^6^/ml) were stimulated with PMA 10 and ionomycin 50 ng/ml, or PMA 50 and ionomycin 100 ng/ml for the times indicated. Cells were harvested and washed in ice-cold PBS. Cytoplasmic and nuclear extracts were prepared by a method adapted from Dean *et al* [44]: 5×10^6^ cells were resuspended in 500 µl hypotonic lysis buffer (10 mM HEPES pH 7.6, 40 mM KCl, 3 mM MgCl_2_, 5% glycerol, 1% Triton X-100, 2 mM DTT, 2 mM NaF, 1 mM Na_3_VO_4_, 25 mM β-glycerophosphate and protease inhibitor cocktail 10 µl/ml) for 5 minutes on ice. After centrifugation at 500 *g* x 3 minutes at 4 °C, the supernatant was saved (cytosolic fraction), and pelleted nuclei washed in 250 µl wash buffer (10 mM HEPES pH 7.6, 10 mM KCl, 1.5 mM MgCl_2_, 1 mM DTT, 2 mM NaF, 1 mM Na_3_VO_4_, 25 mM β-glycerophosphate and protease inhibitor cocktail 10 µl/ml). A sample was examined for successful nuclear extraction of intact nuclei by light microscopy. Nuclei were pelleted as before then extracted in 150 µl nuclear extraction buffer (20 mM HEPES pH 7.6, 420 mM NaCl, 1.5 mM MgCl_2_, 25% glycerol, 1 mM DTT, 0.2 mM EDTA, 2 mM NaF, 1 mM Na_3_VO_4_, 25 mM β-glycerophosphate and protease inhibitor cocktail 10 µl/ml) for 15 minutes on ice, subjected to one freeze-thaw cycle at −80 °C, and clarified by centrifugation at 14,000×*g* for 15 minutes at 4 °C. Nuclear extract and cytosol were stored at −80 °C until use. For immunoblot, up to 20 µg nuclear or cytosolic proteins were separated by SDS-PAGE (NuPAGE Bis-Tris MOPS system, Invitrogen) and proteins of interest detected by immunoblot and ECL (GE Healthcare Life Sciences, Amersham, UK). Immunoblots of all nuclear and cytoplasmic proteins were probed for β-actin, which is abundant in both nucleus and cytoplasm [45], [46], and α-tubulin, abundant in cytoplasm only. Actin detected in the absence of tubulin indicated freedom of nuclear extracts from cytosolic contamination and was used to normalise for protein loading in both nuclear and cytosolic immunoblots. For EMSA, 5 µg nuclear proteins were incubated with ^32^P end-labelled oligonucleotide probe for 15 minutes at room temperature then separated in a 6% non-denaturing polyacrylamide gel, the gel dried, and protein-bound oligonucleotide detected by phosphor-imaging and by exposure of photographic film to dried gel. For probe competition, cold probe was added to the incubation in 100-fold excess. For super-shifting assays, proteins were incubated with 1 µg antibody for 10 minutes at room temperature prior to addition of radiolabelled probe. For immunoprecipitations, 5×10^6^ cells were lysed in 500 µl 50 mM TRIS.HCl pH 8, 200 mM NaCl, 1% TX-100, 1 mM DTT, 0.1 mM EDTA, 1 mM Na_3_VO_4_, 50 mM NaF, 10 mM β-glycerophosphate, and protease inhibitor cocktail 10 µl/ml. IκB and NF-κB proteins were immunoprecipitated from 100 µg clarified lysate and analysed by immunoblot for the target and co-immunoprecipitated proteins.

### IκBε−/− mice

The *IκBε* coding sequence was replaced by an *nlslacZ/IRESneo* construct and fully back-crossed onto the C57BL/6J background as reported previously by Mémet and colleagues [47]. Mice were bred and housed in a specific pathogen-free environment and genotyped by PCR, as described (30). IκBε +/+ wild-type, +/− heterozygote, or −/− homozygote knockout (>7 weeks or <18 weeks old) male mice were used in all experiments unless otherwise stated. Where WT mice were not littermates of IκBε−/− animals, they were age-matched to within 2 weeks and housed beside the mutant animals for at least 14 days prior to experimentation. Mice were killed by cervical dislocation or asphyxiation with CO_2_, and spleen and lymph nodes (submandibular, axillary, inguinal, popliteal, mesenteric and para-aortic) dissected, from which splenocytes and lymph node cells (LNC) were prepared.

### Generation of primary T cell blasts, T cell stimulation, nuclear and cytoplasmic extracts and immunoblotting; assay of intracellular IL-2

Mixed splenocytes and LNC were cultured at 8×10^6^ cells/ml in complete medium containing anti-CD3ε 100 ng/ml at 37 °C and 5% CO_2_. After 48 hours, cells were washed and resuspended to the same cell density in fresh medium containing recombinant murine IL-2, 20 units/ml. After a further 48 hours, fresh medium and IL-2 were added, to the same final concentration. Three days later, T cell blasts were washed and restimulated with plate-bound anti-CD3ε 5 µg/ml (5×10^6^ cells per sample) for up to 4 hours at 37 °C and 5% CO_2_, before harvesting on ice, by scraping, and preparation of nuclear and cytosolic extracts as above (per 5×10^6^ cells: 150 µl hypotonic lysis buffer, 75 µl wash buffer, 40 µl nuclear extraction buffer). 15 µg cytosolic proteins, or total nuclear extracts were assayed for NF-κB and IκB proteins by immunoblot. Relative expression of nuclear and cytosolic proteins was calculated from quantitative densitometric scans (GS710 Calibrated Imaging Densitometer with Quantity One software [Bio-Rad Laboratories, Hemel Hempstead, UK]) using the Phoretix 1D software (Non-Linear Dynamics, Newcastle-upon-Tyne, UK). In parallel experiments, cells (2.5×10^5^ cells per point) were stimulated with PMA 10 ng/ml and ionomycin 500 ng/ml in the presence of brefeldin A 10 µg/ml for 6 hours at 37 °C and 5% CO_2_. Cells were then washed and labelled with anti-CD4-PE-Cy7 and anti-CD8-PE labelled antibodies, both at 1∶100 in FACS buffer (2% BSA, 0.02% sodium azide in PBS), for 20 minutes at 4 °C in the dark. Surface-labelled cells were washed and fixed in paraformaldehyde (2%) for 10 minutes at room temperature, washed, permeabilised with saponin 2% in FACS buffer, and labelled with anti-IL-2-FITC 1∶50 in permeabilisation buffer for 1 hour at 4°C in the dark. Labelled cells were washed and flow cytometry data acquired in a BD^TM^ LSR II flow cytometer prior to analysis using FACSDiva™ software (BD Biosciences).

### Thymidine incorporation assay

Splenocytes or LNC were plated at 5×10^4^ cells per well in 200 µl complete medium and stimulated for 48 hours with soluble anti-CD3ε and anti-CD28 at a range of concentrations at 37 °C and 5% CO_2_. 100 µl conditioned medium was then removed from each well, for cytokine analysis, and 1 µCi ^3^H-thymidine added. After 18 hours' further incubation, ^3^H-thymidine incorporation was measured in a MicroBeta JET plate-reader (Perkin Elmer, Buckinghamshire, UK).

### Lymphocyte phenotyping and B cell selection and survival

Splenocytes, LNC, or B and T cell numbers and viability were assayed by trypan blue exclusion under light microscopy or by flow cytometry (FSc/SSc live gate). Cell phenotypes were analysed by flow cytometry after labelling with anti-B220-FITC, anti-CD11b-APC and anti-CD4/CD8 antibodies as above. B cells were prepared from spleens by positive selection using anti-CD19-coated magnetic beads according to the manufacturer's instructions (Miltenyi Biotech Ltd, Bisley, UK). B cells - more than 90% pure by B220-FITC labelling - were cultured without antigen receptor stimulation at 10^6^ cells per well (per ml) in 24 well plates for 48 hours at 37 °C and 5% CO_2_ and assayed daily by flow cytometry for viability and receptor expression using either anti-B220-FITC plus anti-CD40-APC, or anti-BAFF-R-FITC plus anti-CD40-APC. In other experiments, purified B cells were cultured as above but in the presence of 10 µg/ml anti-IgM F(ab')_2_ fragment (Jackson ImmunoResearch Europe, Ltd, Newmarket, UK ) plus 2 units/ml mIL-4 (R&D Systems, Oxford, UK) for up to 72 hours and assayed daily by flow cytometry for viability and receptor expression using anti-B220-FITC plus anti-CD40-PE and anti-BAFF-R-APC.

### B cell stimulation, nuclear and cytoplasmic extracts and immunoblotting

Splenic B cells were prepared as above from 3 age- and sex-matched WT and IκBε−/− mice plated at 10^7^ cells per ml in a 12-well plate in complete medium at 37 °C and 5% CO_2_, and allowed to recover for 2 hours. Cells were then stimulated (10^7^ per condition) for 0, 0.5 and 1 hour with 10 µg/ml anti-IgM F(ab')_2_ fragment, and nuclear and cytoplasmic extracts prepared as for T cells. Total nuclear extracts or 10 µg cytosolic proteins were immunoblotted for c-Rel, p65, NFAT2, c-Fos, IκBε and -α and actin. Detection was by chemiluminescence in a BioRad Chemi Doc™ XRS+ Molecular Imager® and c-Rel expression relative to actin expression analysed with Image Lab™ software.

## Results

### TNF inhibits IL-2 gene transcription and attenuates induction of AP-1 and NFAT

**Figure 1:**
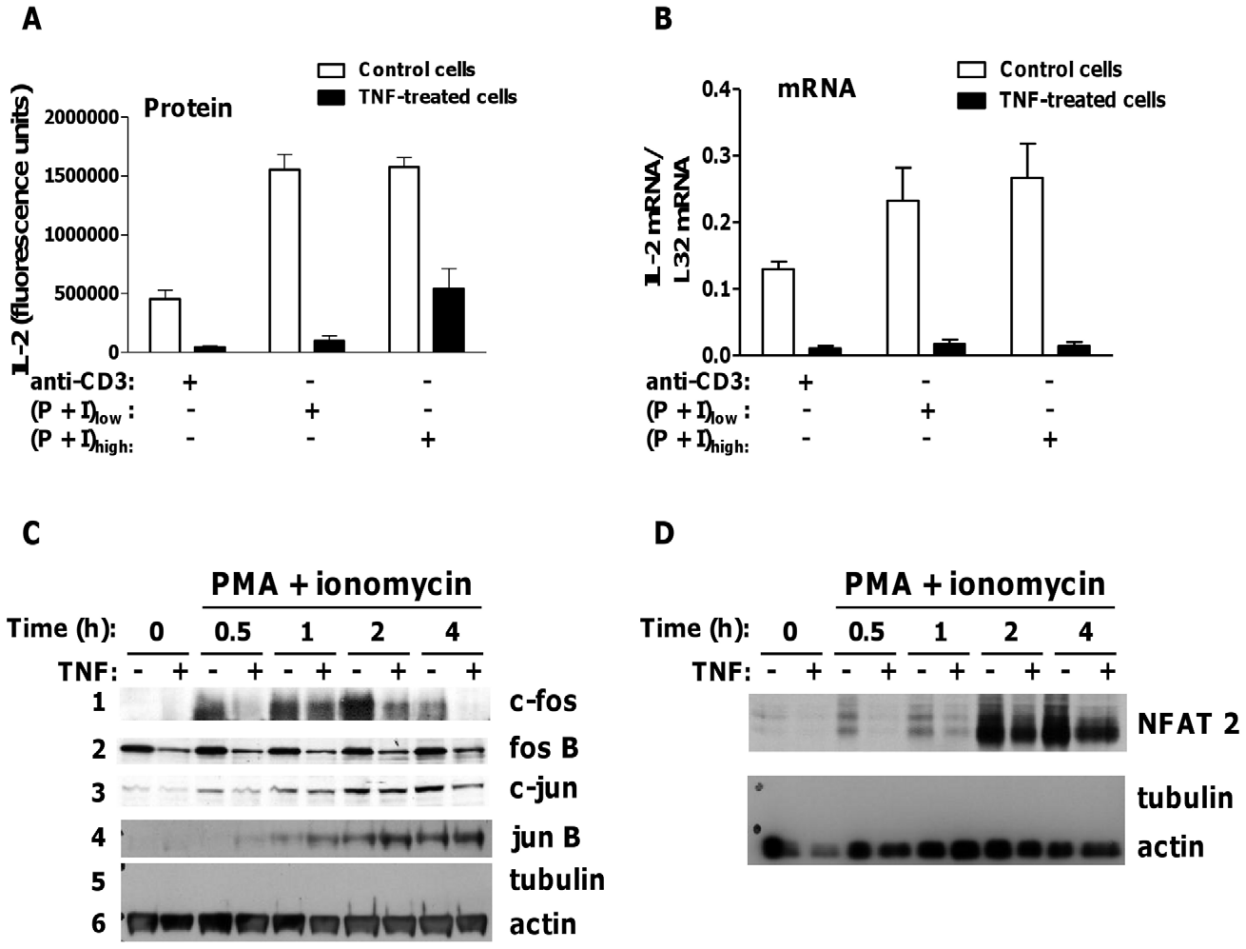
TNF inhibits IL-2 gene transcription and attenuates induction of AP-1 and NFAT. 11A2 cells were cultured with or without TNF 2.5 ng/ml for 8 days before (A) restimulation with plate-bound anti-CD3ε 5 µg/ml, PMA 10 ng/ml and ionomycin 50 ng/ml (P+I)_low_, or PMA 50 ng/ml and ionomycin 100 ng/ml (P+I)_high_ for 24 hours. Secreted IL-2 protein was measured by immunoassay (mean +/− SEM, n = 5 experiments); (B) restimulation as above for 4 hours followed by RNA extraction, and analysis for IL-2 mRNA by RNase protection assay (mean +/− SD, n = 3 experiments). After restimulation with (P+I)_low_, nuclear and cytoplasmic extracts were prepared, and equivalent amounts of protein assayed by immunoblot for (C) nuclear AP-1 and (D) nuclear NFAT2 (representative of 3 experiments for each condition). Each blot was probed then reprobed for the indicated proteins.

When studying effects of TNF on T cell function, we observed that pre-treatment of mouse T cell hybridoma 11A2 with picomolar concentrations of TNF suppressed induction of secreted IL-2 protein by more than 90%, relative to untreated cells. Major suppression occurred whether T cells were activated via TCR or with two non-toxic concentrations of PMA and ionomycin, being partly recovered when the higher of the two was used (P+I)_high_, and was therefore independent of any attenuation of TCR-proximal signalling that we have reported previously (Figure 1A and [41], [42]). Inhibition of IL-2 induction was at the level of mRNA expression, as determined by ribonuclease protection assay, peak expression of IL-2 mRNA being reduced by at least 90% in TNF-treated as compared to control cells, for all stimuli (Figure 1B). We tested the effect of TNF on stability of induced IL-2 mRNA and found that, for cells stimulated with PMA plus ionomycin, IL-2 mRNA was stable for up to 3 hours after addition of actinomycin D, even though its peak expression in TNF-treated cells was <10% that of control cells (Figure S1, shown for (P+I)_low_ ). This indicated that inhibition of IL-2 induction in TNF-treated cells was not due to reduced mRNA stability. Therefore, we focussed our studies on the effects of TNF on transcriptional regulation of IL-2.

The IL-2 proximal promoter (pIL-2) contains simple and composite binding sites for NFAT, AP-1 (Fos/Jun) and NF-κB transcription factors. We compared induction and nuclear translocation of AP-1, NFAT and NF-κB proteins in control and TNF pre-treated cells by immunoblotting. AP-1 exists as homodimers of jun (c-Jun, Jun B, Jun D) or heterodimers of Fos (c-Fos, Fos B, Fra-1 and Fra-2) and Jun. Induction of nuclear c-Fos by PMA and ionomycin was rapid and transient in control 11A2 cells, being detectable at 30 minutes, peaking at 2 hours and diminishing at 4 hours. In TNF-treated cells, by contrast, its induction was delayed and more transient (Figure 1C, panel 1). Nuclear Fos B was also attenuated in TNF-treated cells as was, to a lesser extent, c-Jun (panels 2 and 3). However, inducible nuclear Jun B was unaffected by TNF (panel 4). Fra-1 and Fra-2 were not detected consistently by immunoblot. In resting primary T cells, hyper-phosphorylated NFAT is bound to inactive calcineurin phosphatase in the cytoplasm. A sustained calcium signal activates calcineurin, leading to dephosphorylation and nuclear translocation of NFAT. NFAT1 is constitutively expressed in T cells whereas NFAT2 is inducible. In 11A2 cells, dephosphorylated NFAT1 was expressed constitutively in the nucleus, to similar levels in control and TNF-treated cells (Figure S2). Induced nuclear NFAT2 was detected at two hours, with levels increasing after 4 hours' stimulation (Figure 1D). Its nuclear expression was lower and slightly delayed relative to control cells, but NFAT2 was nonetheless strongly induced, dephosphorylated and imported to the nucleus in TNF-treated cells.

### TNF pre-treatment inhibits subsequent induction and nuclear expression of NF-κB associated with upregulation of IκBε

**Figure 2:**
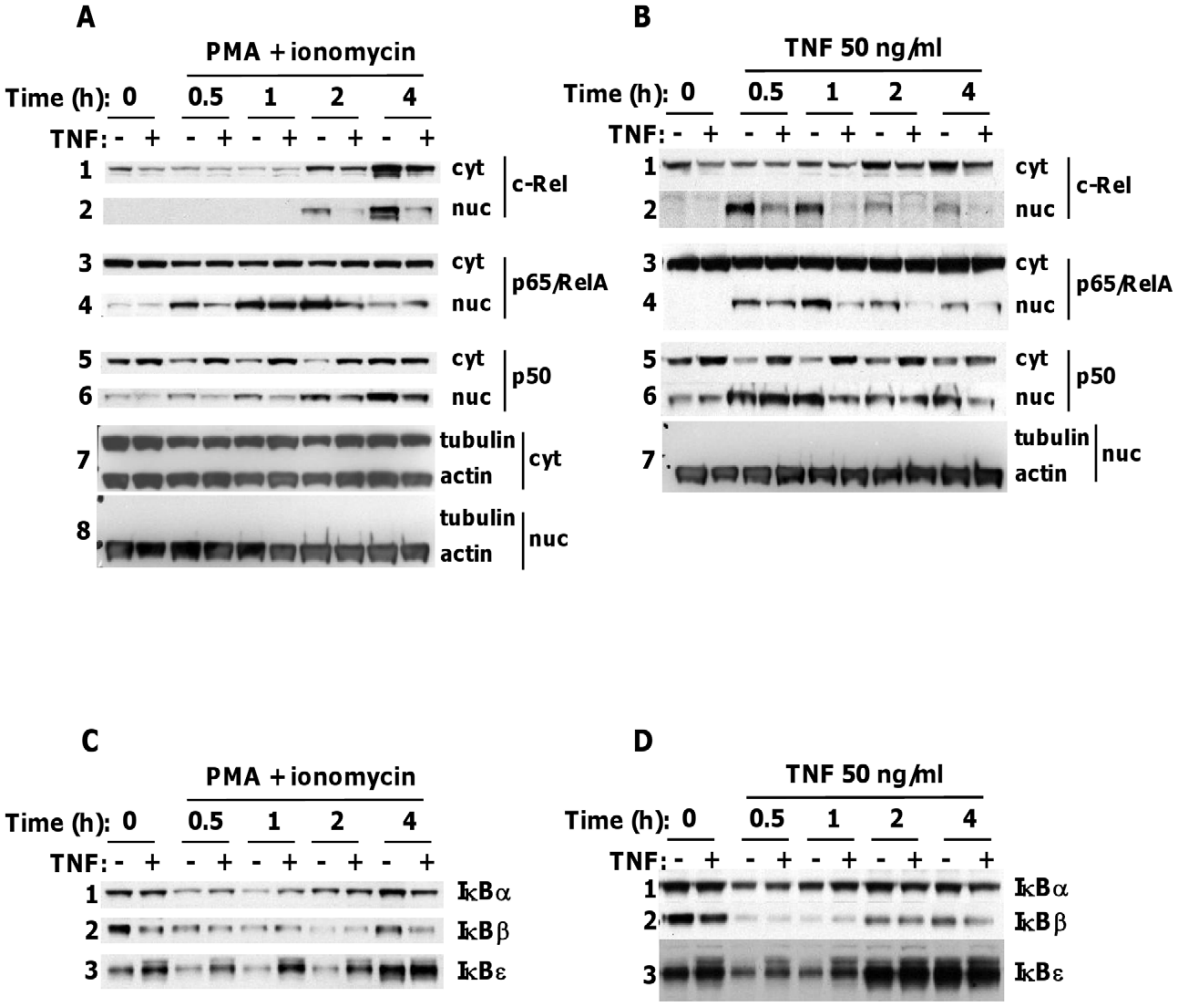
TNF pre-treatment inhibits subsequent induction and nuclear expression of NF-κB and upregulates IκBε. 11A2 cells were cultured with or without TNF 2.5 ng/ml for 8 days before restimulation with PMA 10 ng/ml and ionomycin 50 ng/ml or TNF 50 ng/ml for the time-points indicated. Nuclear and cytoplasmic extracts were prepared and equivalent amounts of protein assayed for the presence of NF-κB transcription factors (A, B) and IκB isoforms (C, D) by immunoblot. Each blot represents at least three similar experiments, probed then reprobed for the indicated proteins.

p65 and c-Rel were detected in the cytosols of resting control and TNF-treated 11A2 cells (Figure 2A, panels 1, 3). Newly-synthesised c-Rel appeared in the cytosol of control and, to a lesser extent, TNF-treated cells after 1–2 hours' stimulation with PMA and ionomycin (Figure 2A, panel 1). However, nuclear c-Rel, detectable at 2 hours, was greatly reduced in TNF-treated relative to control cell samples (panel 2) and remained barely detectable at 6 or 8 hours (not shown). TNF-treated cells also showed a reduction in nuclear translocation of both p65 (panel 4) and p50, more p50 being retained in the cytosol of TNF-treated compared to control cells (panels 5, 6).

To test whether the inhibitory effects of TNF on NF-κB signalling were limited to the TCR pathway, whose activation is mimicked by PMA and ionomycin, we analysed nuclear and cytosolic NF-κB in control and TNF-pre-treated 11A2 cells following acute restimulation with TNF (50 ng/ml). Acute TNF stimulation led to rapid, transient nuclear translocation of c-Rel, p65 and p50 which peaked between 30 minutes and 1 hour of stimulation (Figure 2B, panels 2, 4, 6). However, c-Rel was barely detectable in nuclear extracts of TNF-pre-treated cells at any time-point of acute TNF stimulation (panel 2). By contrast, acute TNF-induced nuclear translocation of p65 and p50 was only slightly reduced at most time-points tested (panels 4, 6), although more p50 remained in the cytosol of TNF-pre-treated cells at all time-points, compared to control cells (panel 5). Thus, pre-treatment of 11A2 cells with TNF also inhibited TNF-R-induced NF-κB nuclear translocation, most markedly that of c-Rel. These data suggested that the effects of TNF pre-treatment on NF-κB activation were not restricted to TCR/CD28-dependent pathways involved in IL-2 gene expression. Rather, TNF attenuated NF-κB activity by modulating a receptor-distal factor common to TCR- and TNF-R-dependent NF-κB activation, IκB being a good candidate.

We therefore examined inducible degradation of IκBs -α, -β and -ε by PMA and ionomycin (Figure 2C) or TNF re-stimulation (Figure 2D), in control and TNF-treated cells. TNF pre-treatment led to increased basal levels of IκBε, but not of -α or -β (Figure 2C, D: compare first and second lanes). IκBs -α and -β were rapidly degraded to similar degrees in control and TNF-treated cells in response to either stimulus (Figure 2C, D; panels 1 and 2) and were re-expressed after 1–2 hours (α), or 2–4 hours (β), of stimulation, re-expression of IκBβ being more marked in control than TNF-treated cells. By contrast, IκBε was present at increased levels in TNF-treated compared to control cells for stimulations of up to 2 hours. IκBε also appeared resistant to PMA and ionomycin-induced degradation (compare 0.5 and 1 hour time-points of control and TNF-treated cells, Figure 2C, D, panels 3), and returned to unstimulated cell levels more rapidly in TNF pre-treated than in control cells (compare 0 and 1 hour time-points of control and TNF-treated cells, Figure 2C, D; panels 3). Thus, both inhibition of nuclear translocation of NF-κB, particularly of c-Rel, and sustained, increased expression of IκBε in TNF pre-treated cells were evident upon acute activation TCR and TNF-R pathways.

### Transcription factor DNA binding and activity are suppressed by TNF: transcriptional activity partially restored by exogenous c-Rel

**Figure 3:**
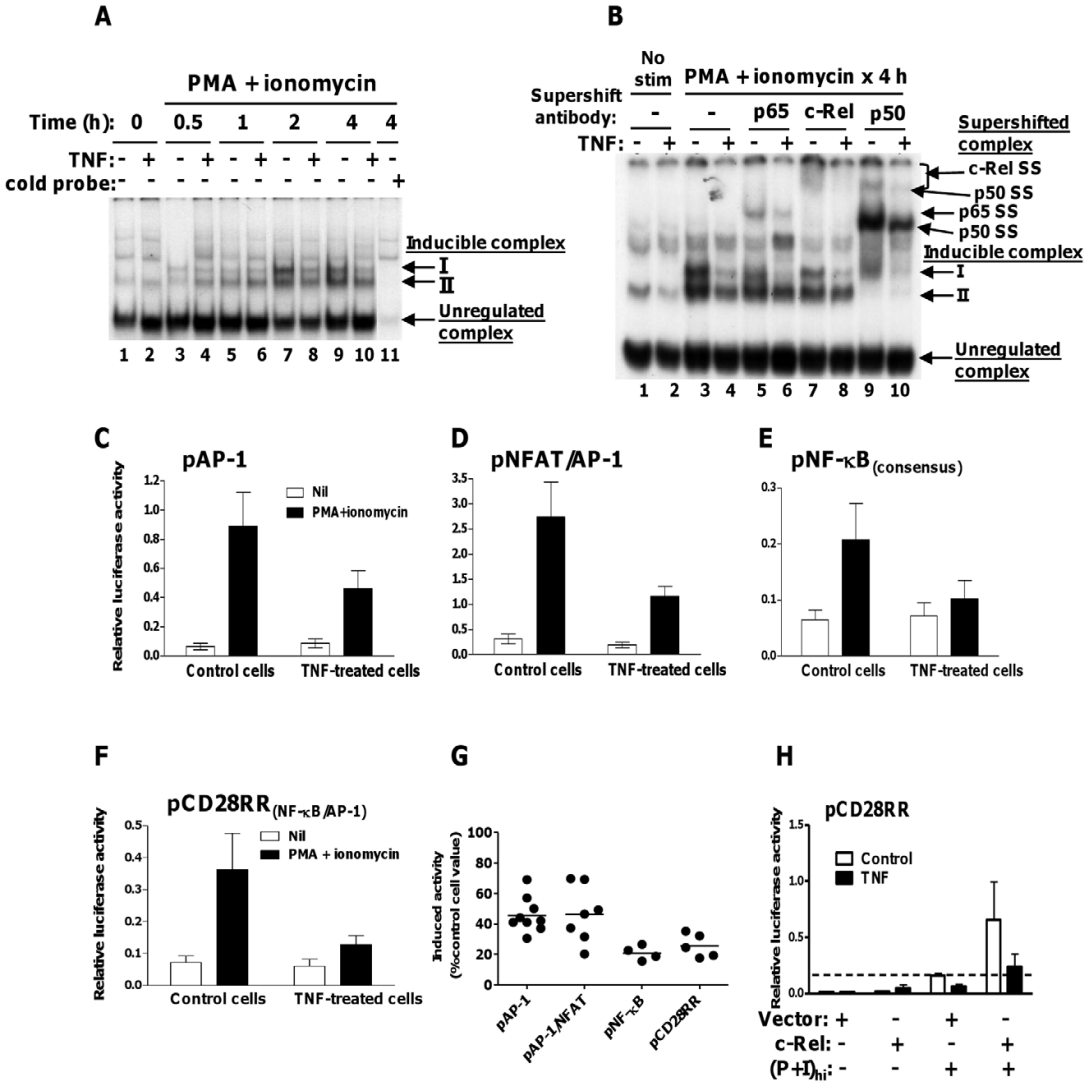
Transcription factor DNA binding and activity are suppressed by TNF. Activation of pCD28RR partially restored by exogenous c-Rel. Control and TNF-treated 11A2 T cells were activated with (P+I)_high_. Nuclear extracts were incubated with (A) ^32^P-labelled NF-κB consensus oligonucleotide or cold competitor probe or (B) ^32^P-labelled NF-κB oligonucleotide with or without NF-κB supershifting antibodies, before non-denaturing gel electrophoresis. Protein-bound oligonucleotide was visualised by both phosphorimaging and exposure of the dried gel to photographic film. Images are representative of three experiments. Control and TNF-treated cells were transfected with reporter plasmids (10 µg) expressing firefly luciferase under control of (C) pAP-1, (D) pNFAT/AP-1, (E) pNF-κB_consensus_, (F) pCD28RR_(NF-κB/AP-1)_ promoters, and stimulated as above for 6 hours. Firefly luciferase expression relative to pSV40-driven renilla luciferase from a co-transfected plasmid (0.2 µg) is shown (mean +/− SEM for 9, 7, 4 and 5 experiments, respectively). (G) Summary of inducible promoter activities in TNF-treated cells as a percentage of induction in control cells, for all experiments. (H) Control and TNF-treated 11A2 cells were cotransfected with 2 µg of empty vector or c-Rel expression plasmid and pCD28RR reporter plus renilla luciferase plasmids prior to stimulation as for F. The dotted line represents stimulated expression in cells transfected with empty vector (mean +/− SEM, n = 4 experiments).

We used electrophoretic mobility shift assay (EMSA) to test whether reduced nuclear levels of NF-κB, AP-1 and NFAT proteins in TNF-treated 11A2 cells were reflected by attenuated DNA-binding. Inducible binding to radiolabelled NF-κB consensus oligonucleotide was detected at 2 hours', and increased after 4 hours' stimulation with PMA and ionomycin in control cell extracts (Figure 3A, lanes 7 and 9). Two inducible protein/DNA complexes (upper complex I, lower complex II) were observed. At these time points, which correspond to those for induction and nuclear translocation of c-Rel by PMA and ionomycin, the intensity of signal due to both complexes, particularly complex I, was lower in TNF-treated relative to control cell samples (Figure 3A, compare lanes 7 and 8, and 9 and 10). Analysis of NF-κB in complexes I and II following 4 hours' stimulation of cells, using subunit-specific antibodies, revealed that complex II appears to contain chiefly p50 protein (Figure 3B, lanes 9 and 10), and complex I, p65, c-Rel and p50 (lanes 5–10). Markedly less c-Rel, p65 and p50 were detected in inducible complexes I and II derived from TNF-treated, as compared to control cell samples (compare lanes 4, 6, 8 and 10 with lanes 3, 5, 7 and 9). This difference in DNA-bound NF-κB was greatest for complex I, consistent with the more marked TNF-dependent reduction in nuclear c-Rel and p65 relative to p50 already observed.

Maximal inducible AP-1 protein binding to radiolabelled probe was also greater in control compared to TNF-treated cell nuclear extracts. Subunit-specific supershift indicated modest reductions in DNA bound c-Fos and Jun B in TNF-treated cell extracts compared to controls, but little or no difference in the amounts of Fos B, Fra-2, c-Jun, or Jun D (Figure S3B, C; c-Jun not shown; Fra-1 not detected). EMSA and supershift with anti-NFAT2 antibody revealed similar amounts of NFAT2 in inducible NFAT/AP-1 binding complexes from control or TNF-treated cell samples, despite reduced complex size for the latter (Figure S3D). However, since reduced NFAT/AP-1 complex size in TNF-treated cell samples was consistent in three experiments but the supershift worked well only in the assay shown, the discrepancy between antibody-supershifted and unshifted complex sizes may be unique to this particular experiment. NFAT1 which was constitutively nuclear in 11A2 cells and expressed to similar levels in control and TNF-treated cells (Figure S2) was not assayed for NFAT/AP-1 DNA binding.

In parallel experiments, we tested the effect of reduced nuclear levels and attenuated DNA-binding of these transcription factors on the ability of cells to activate promoters containing consensus sequences for NF-κB, AP-1 and NFAT. The two composite sequences in pIL-2 which have been shown to be important for gene activation are an NFAT/AP-1 site at position −274 to −287 [27], and an NF-κB/AP-1 site also known as the CD28 response region (CD28RR) at position −147 to −163 from the transcription start site [28]; the non-consensus NF-κB-binding element in CD28RR (CD28RE) preferentially binds c-Rel-containing dimers in stimulated T cells [48]. We measured PMA and ionomycin-stimulated expression of firefly luciferase under control of pAP-1, pNFAT/AP-1, pNF-κB, or pCD28RR. Basal and induced firefly luciferase activities were expressed relative to co-transfected constitutive Renilla luciferase expression in the same cell lysate sample (see Methods).

Transcriptional activation was suppressed in TNF-treated cells as compared to control cells, for all promoters tested (Figure 3C–3F), by 55–60% for pAP-1 and pNFAT/AP-1, 80% for pNF-κB and 75% for pCD28RR (Figure 3G). These data correlate with the greater attenuation which we observed in nuclear expression and DNA-binding of NF-κB, particularly c-Rel, relative to NFAT and AP-1, in TNF-treated cells. We therefore tested the ability of exogenous c-Rel to overcome suppressed activation of pCD28RR in TNF-treated T cells by co-transfecting c-Rel expression vector, or empty vector, with firefly luciferase reporter and constitutive Renilla luciferase plasmids. Co-transfection of TNF-treated 11A2 cells with c-Rel restored inducible activation of pCD28RR to a level equivalent to that of control cells transfected with empty vector (Figure 3H). However, exogenous c-Rel led to a 4-fold increase in inducible pCD28RR activation for control as well as TNF-treated cells, implying firstly that c-Rel concentration is limiting in terms of activation of CD28RR in untreated 11A2 cells and, secondly, that reconstitution of factors other than c-Rel – such as AP-1 subunits – is required for maximal activation of CD28RR in TNF-treated cells.

### PMA and ionomycin-induced c-Rel associates with IκBα and IκBε; persistent and enhanced association of IκBε with p65/c-Rel in TNF-treated cells throughout stimulation

**Figure 4:**
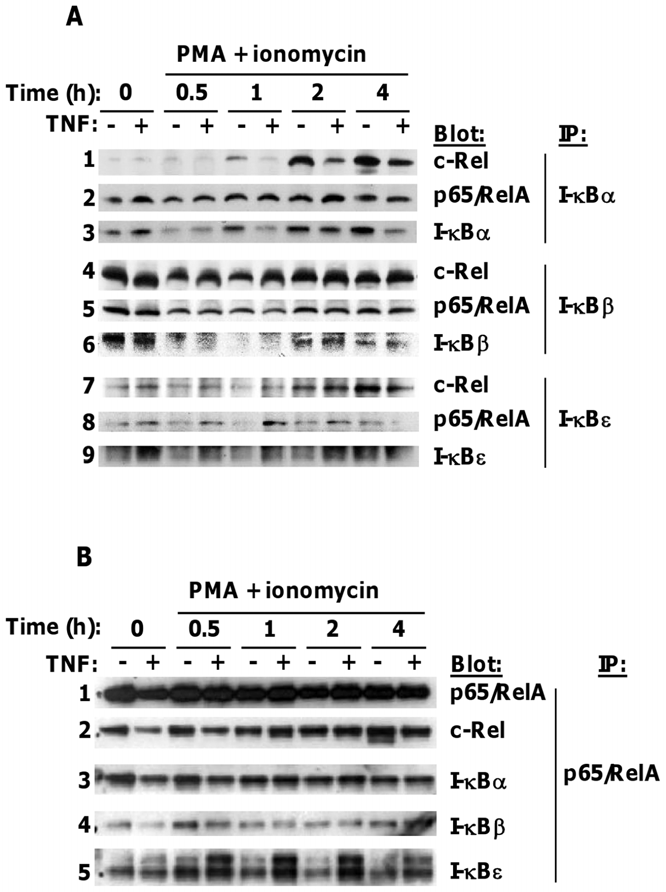
Association of IκBε with newly synthesised c-Rel and p65/c-Rel complexes in TNF-treated cells. Control and TNF-treated 11A2 cells were restimulated with (A) (P+I)_high_, (B) P+I_low_, for the indicated times. Nuclear and cytosolic extracts were prepared. (A) IκBs α, β and ε were immunoprecipitated from 100 µg cytosolic protein and immunoblotted for associated c-Rel and p65. (B) p65-containing complexes were immunoprecipitated from 100 µg cytosolic protein and immunoblotted for associated IκB. Blots were probed and reprobed for the indicated proteins and those shown are representative of more than 3 experiments at both concentrations of P+I.

Our data implied that TNF pre-treatment had inhibitory effects on all three transcription factor families required for optimal activation of the IL-2 promoter. However, we became most interested in the selective increase in IκBε expression, out of the IκBs, accompanied as it was by strong inhibition of c-Rel nuclear translocation, out of all NF-κB subunits tested, in TNF-treated cells. We decided to use this system to investigate the association of c-Rel and IκBε further.

c-Rel has been shown to be chiefly associated with IκBβ in naïve, but with IκBα in TNF- and IL-1-stimulated, T cells [49]. However, the contribution of IκBε was not examined in that study. Biochemical analysis demonstrated that IκBε binds c-Rel and p65 hetero- or homodimers in preference to p65/p50, which tends to be bound by IκBα [8]. We therefore explored the composition of NF-κB/IκB complexes in TNF pre-treated cells following stimulation with PMA and ionomycin. We immunoprecipitated IκBα, -β, or -ε from equal amounts (100 µg) of cytosolic protein, and immunoblotted for associated NF-κB (Figure 4A; see Figure S4A (i) and (ii) for proportions of each protein in cytosol and post-IP supernatants). IκB proteins were precipitated efficiently by their individual antibodies, since little IκB was detected by immunoblot in post-IP supernatants (Figure S4A (i). However, p65 and c-Rel were still detectable in these post-IP supernatants (Figure S4B (ii)) suggesting that only a small proportion of either NF-κB co-precipitated with a single IκB isoform.

In resting cells c-Rel was associated with IκBβ (Figure 4A, panel 4) and p65 was associated with both IκBα and β (Figure 4A, panels 2 and 5). A small amount of c-Rel and p65 also co-immunoprecipitated with IκBε (Figure 4A, panels 7 and 8). The amounts of p65 or c-Rel associated with each IκB were reduced when the IκB was degraded, and increased as the IκB was resynthesised, following stimulation. Newly synthesised c-Rel, expressed after 2 hours, associated with IκBα and IκBε as well as IκBβ (Panels 1, 4 and 7, 2–4 hour time-points). The amounts of p65 associating with IκBα and β, and the amount of c-Rel associating with IκBβ, were similar for control and TNF-treated cell samples (Figure 4A, panels 2, 4 and 5). However, the amount of c-Rel associated with IκBε was greater in TNF-treated cell samples compared to controls up to 2 hours of stimulation (panels 7 and 9: 0–2 hour time-points). This difference was lost when IκBε expression in control cells reached that of TNF-treated cells (panels 7 and 9, 4 hour time-point). Thus the amount of c-Rel immunoprecipitating with IκBε reflected the expression level of IκBε. By contrast, association of newly-synthesised c-Rel with IκBα reflected the greater abundance of c-Rel in control compared to TNF-treated cells (Figure 4A, panels 1 and 3 and Figure S4A (i) and (ii)). In three experiments, it was difficult to obtain very clear bands for immunoprecipitated IκBs -β and -ε, because the signal for these 45 kD proteins ran just in front of the heavy chain band of immunoprecipitating antibody in blots. We were unable to detect p50 in any co-immunoprecipitation experiment.

We also immunoprecipitated p65, using directly-conjugated agarose beads, and blotted for associated IκB proteins. We were unable to immunoprecipitate c-Rel directly by a similar method, but found that a proportion of c-Rel co-precipitated with p65 (Figure 4B, panels 1 and 2). Blots of post-IP supernatants revealed efficient co-immunoprecipitation of IκBs with p65 (Figure S4C). p65 itself was not always pulled down with identical efficiency, as may be seen by variation in immunoprecipitated p65 signal compared to the signal in input cytosol (Figure 4B, panel 1 and Figure S4B, panel 1).

All three IκBs associated with p65/c-Rel complexes. In resting and stimulated cells, the amounts of IκBs -α and -β associated with p65/c-Rel were similar for control and TNF-treated cell samples, once efficiency of the p65 pull-down was taken into account (Figure 4B, panels 3 and 4 – compare with panel1). However, at all time-points, more IκBε was associated with the p65/c-Rel complexes in TNF-treated than in control cell extracts even when expression of IκBε in control cells approached that of TNF-treated cells, at four hours (Figure 4B, panel 5: lanes 9 and 10, compare with Figure S4B panel 5). Taken together, these experiments indicate that IκBε sequesters newly-synthesised c-Rel in proportion to its own expression levels, and exhibits persistent, enhanced association with p65/c-Rel complexes in TNF-treated cells.

To examine whether regulation of IκBε by TNF was a general phenomenon, as opposed to a peculiarity of T cell hybridomas, we cultured murine B cell lines, including A20 and hybridoma SP2/0, as well as primary T cells of Balb/C and C57Bl/6J mice, in the presence or absence of TNF. However, we failed to document TNF-dependent upregulation of IκBε. Indeed, we observed very high levels of IκBε in primary T cell blasts, regardless of the presence of TNF. Therefore, in order to test our hypothesis – of selective regulation of c-Rel by IκBε in primary cells – and explore its functional consequences, we focussed our studies on lymphocytes from IκBε-deficient mice.

### Increased basal nuclear c-Rel and reduced threshold for TCR-dependent proliferation in IκBε null mouse T cells

**Figure 5:**
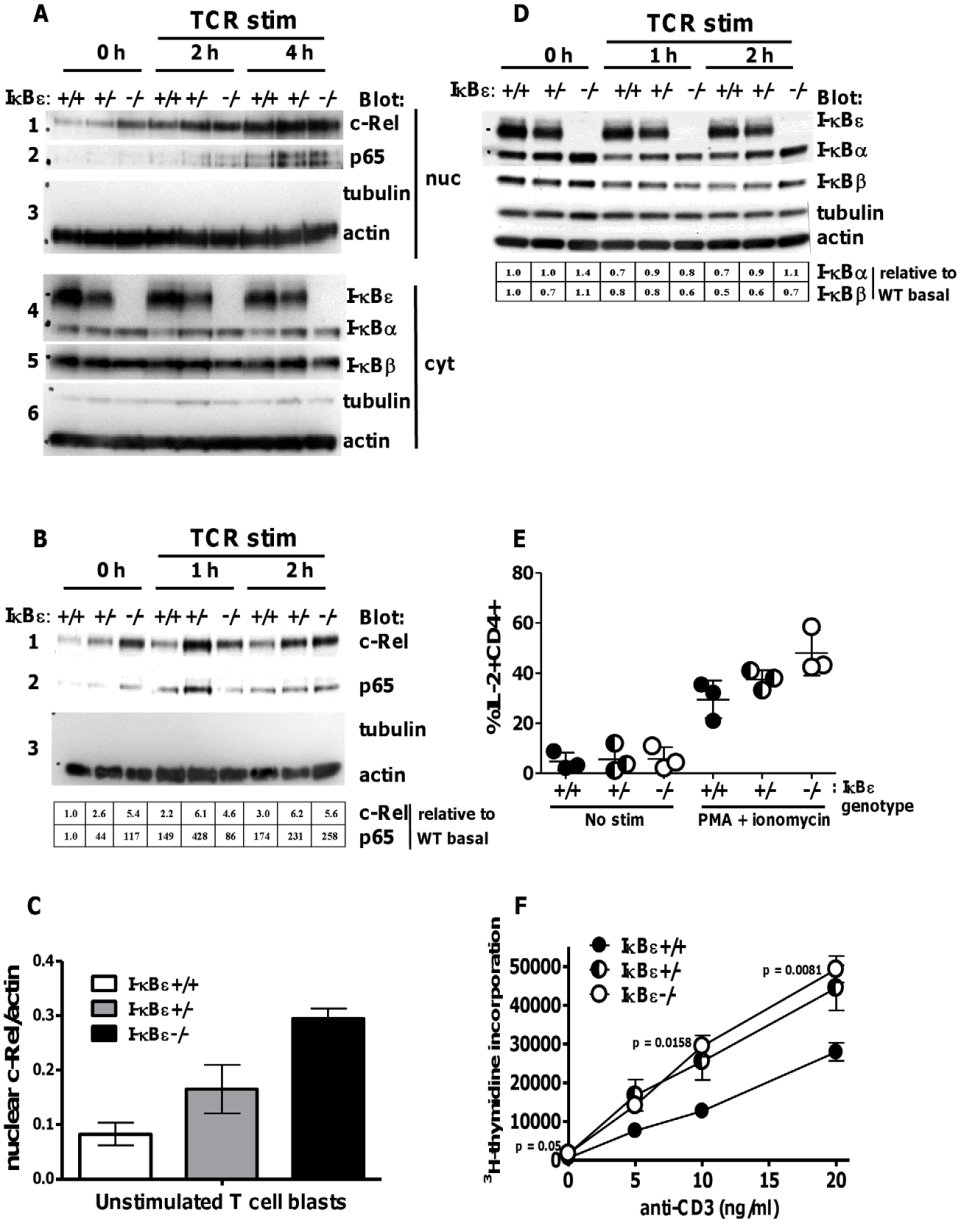
Increased basal nuclear c-Rel, priming for IL-2 expression, and enhanced sensitivity to TCR stimulation in IκBε−/− T cells. Splenocytes and LNCs from WT C57BL/6J (IκBε+/+, filled circles) mice, or mice hetero- (+/−, half-filled circles) or homozygous (−/−, open circles) for IκBε deletion, were cultured with anti-CD3 for 48 hours, washed, then stimulated with IL-2 at day 2 and day 5. On day 7, cells were washed and restimulated, or not, with plate-bound anti-CD3 for up to 4 hours. Extracts from resting and stimulated cells were analysed for (A) nuclear and cytosolic NF-κB and IκB – a four-hour time-course is shown – (B) nuclear NF-κB – a two hour time-course representative of three experiments is shown, with nuclear c-Rel and p65 normalised to actin and expressed relative to values for resting IκBε+/+ T cells as indicated. (C) Relative nuclear expression of c-Rel (mean +/− SD, n = 3 experiments). (D) Cytosolic IκB for experiment shown in (B), with IκBα and -β normalised to actin and expressed relative to values for resting IκBε+/+ T cells as indicated. (E) T cells blasts were stimulated, or not, for 6 hours with PMA and ionomycin in the presence of brefeldin A, labelled with anti-CD4-PECy7, fixed, permeabilised, labelled with anti-IL-2-FITC and analysed by flow cytometry. Shown are% IL-2+CD4+ in the live cell gate (mean +/− SD, n = 3 experiments; one mouse per genotype per experiment). (F) LNC were cultured in triplicate with soluble anti-CD3 at the concentrations indicated. At 48 hours, 1 µCi 3H-thymidine was added to each well and culture continued for 18 hours prior to analysis of 3H-thymidine uptake. Mean +/− SEM, n = 5 experiments (one mouse per genotype per experiment); p values refer to IκBe−/− vs IκBε+/+, other P values were not significant.

We looked first at c-Rel nuclear expression in murine primary T cell blasts, as a correlate of experiments in the 11A2 T cell line. Splenocytes and lymph node cells (LNC) from age- and sex-matched wild-type (+/+), heterozygote (+/−) or homozygous IκBε null (−/−) mice were cultured for 48 hours in the presence of anti-CD3 and expanded for 6 days in IL-2. T cell blasts were then re-stimulated for up to 4 hours with plate-bound anti-CD3 and analysed by immunoblot for NF-κB and IκB proteins. We detected increased nuclear c-Rel in resting T cell blasts from both IκBε+/− and IκBε−/− mice when compared to IκBε+/+, and observed a negative correlation between IκBε gene dose and nuclear c-Rel (Figure 5A and B, panels 1, 0 h time-points). This difference in nuclear c-Rel was most marked for resting T cells and decreased with time of TCR-restimulation until, by four hours' TCR ligation, similar amounts of nuclear c-Rel were detected for all genotypes (Figure 5A, panel 1). Relative band intensities due to c-Rel normalised to nuclear actin in resting T cells are summarised for one experiment in Figure 1B and for all three experiments in Figure 5C and Table S1. Tubulin was not detected in the nuclear extracts indicating that they were free of cytosolic contamination (Figure 5A and B, panels 3). Nuclear p65 was detected at lower levels overall in resting T cells than was c-Rel, and any increase due to IκBε-deficiency was not consistently observed (Figure 5A and B, panels 2 and Table S1); nuclear p50 was not detected. We also observed increased basal IκBα expression in resting cells from IκBε−/− mice (Figure 5A, panel 4, Figure 5D, and Table S1), as has been described [47].

c-Rel was detected in nuclei of resting IκBε−/− T cells despite increased expression of IκBα and normal levels of IκBβ, indicating that IκBε has a non-redundant role in the inhibition of c-Rel nuclear translocation. We wondered whether T lymphocytes would be primed for c-Rel-dependent upregulation of IL-2 in IκBε−/− mice. We found that, although PMA and ionomycin-induced IL-2 expression showed an inverse correlation with IκBε gene dose (Figure 5E), the effect was not statistically significant, and there was no genotype-specific difference in IL-2 mRNA expression by qRT-PCR, in T cell blasts stimulated with plate-bound anti-CD3 (not shown). Further, there was no detectable genotype-dependent difference in the amount of IL-2 secreted following TCR-stimulation of naïve splenocytes or LNC, or T cell blasts. By contrast, when we measured ^3^H-thymidine incorporation by naïve splenocytes and LNC in response to soluble anti-CD3, we found that IκBε+/− and −/− cells appeared more sensitive to stimulation with concentrations of anti-CD3 of less than 20 ng/ml than did WT cells, but the effect was statistically significant only for IκBε−/− cells (Figure 5F). Anti-CD28 alone had no stimulatory effect and did not co-stimulate at concentrations below 1 µg/ml.

### Increased basal thymidine uptake in IκBε−/− cells and increased cellularity of IκBε−/− lymph nodes

**Figure 6:**
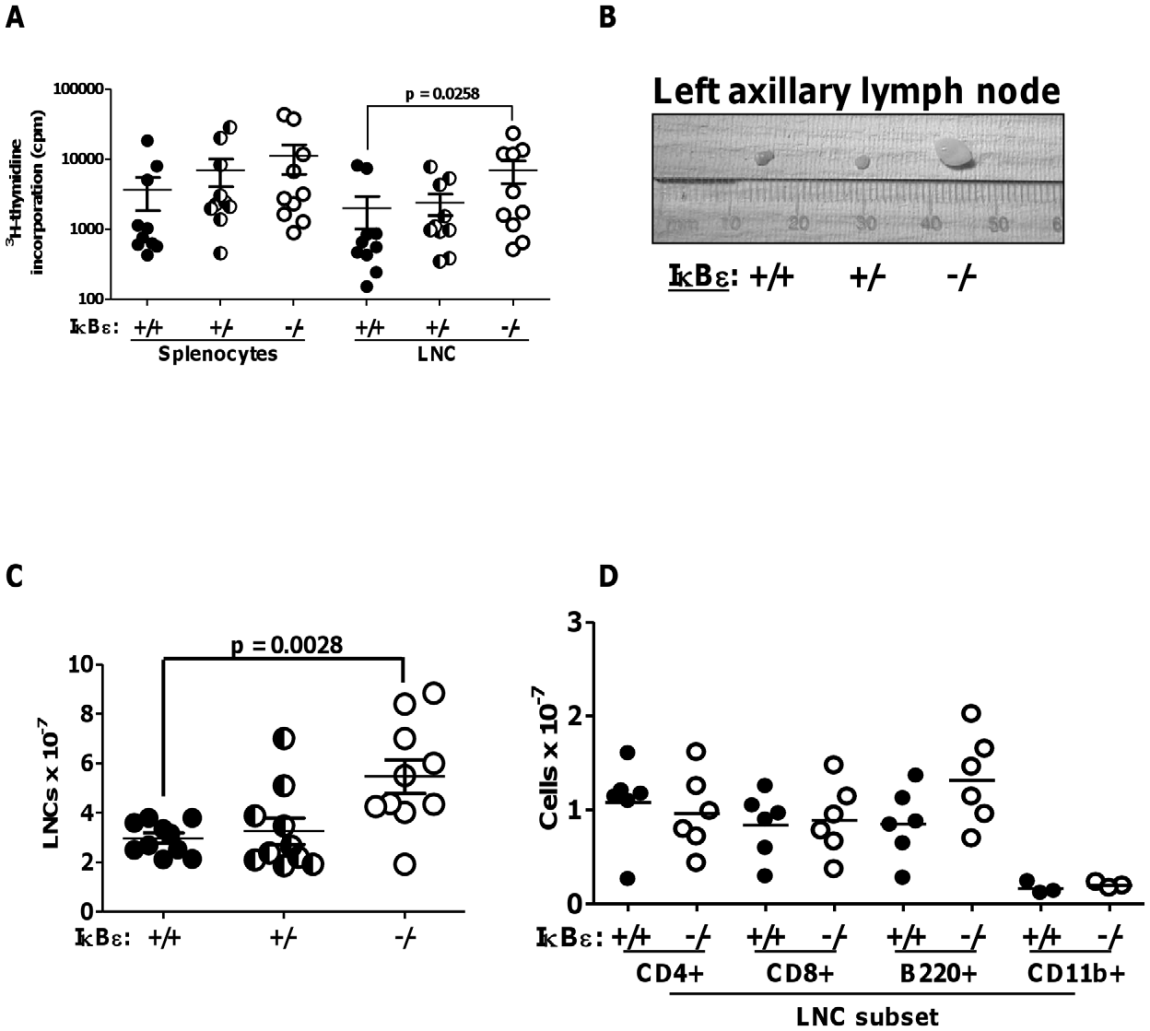
Increased basal thymidine uptake in splenocytes and LNC, increased LN cellularity and B cell numbers in IκBε−/− mice. (A) Basal thymidine uptake by splenocytes and LNCs in IκBε+/+, +/− and −/− mice (median, n = 10 experiments). (B) Enlarged axillary lymph node from an IκBε−/− mouse compared to those from IκBε+/+ and IκBε+/−. (C) Total lymph node cell numbers, one mouse per IκBε genotype per experiment (mean +/− SEM, n = 10). (D) LNC were labelled with anti-CD4-PECy7, anti-CD8-PE, anti-B220-FITC and anti-CD11b-APC before flow cytometric analysis. The percentage of each cell subset was multiplied by the total LNC number to give numbers for each cell subset (mean, n = 6 experiments).

Increased basal thymidine incorporation by IκBε−/− and IκBε+/− cells relative to WT cells was observed consistently for splenocytes, LNC and T cell blasts, and was statistically significant for IκBε−/− LNC (Figure 6A and 5F). However, this increase was not due to c-Rel-dependent basal expression of IL-2 in unstimulated cells, since it was not inhibited with blocking antibodies to IL-2R (data not shown). We also noted that LN were often larger in IκBε−/− compared to WT mice (Figure 6B). Furthermore, total numbers of LN cells, but not splenocytes, were significantly higher for IκBε−/− mice compared to heterozygote or WT (Figure 6C). Phenotypic analysis revealed a trend towards increased numbers of B220^high^ (mature B) cells in the LN of IκBε−/− mice (Figure 6D). We wondered whether increased cellularity of lymph nodes, increased B cell numbers and enhanced basal and TCR-stimulated thymidine uptake by LNC might be explained by c-Rel dependent effects on lymphocyte survival in IκBε−/− mice. We therefore determined expression of the pro-survival co-stimulatory molecule CD40 in WT and IκBε−/− T cell blasts by flow cytometry. Very few CD40+ T cells were detected. However, we found that a population of CD4^−^CD8^−^CD40^+^ cells, which persisted in day 8 cultures of IκBε−/−, but not WT, T cell blasts, were B220+ B cells (not shown). That is, IκBε-/− B cells were surviving longer than WT B cells under conditions favouring T cell expansion. We therefore focussed our studies of IκBε/c-Rel-dependent regulation of lymphocytes on B cells.

### Increased basal nuclear c-Rel and enhanced BCR-stimulated c-Rel translocation in naïve IκBε−/− B cells

**Figure 7:**
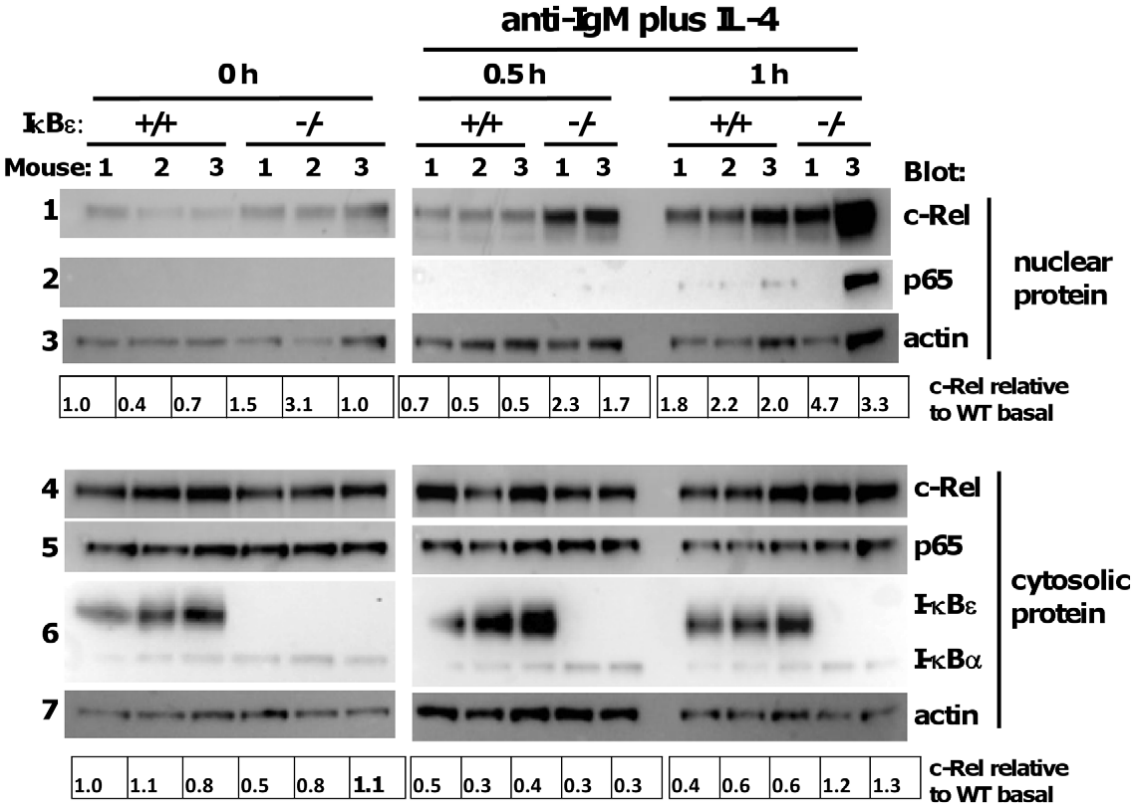
Increased basal and BCR-inducible nuclear c-Rel in IκBε−**/**− splenic B cells. Splenic B cells were isolated by CD19 positive selection from three each of IκBε+/+ and IκBε−/− mice. (A) Cells were rested for 2 hours then stimulated with anti-IgM F(ab')_2_ fragment 10 µg/ml for the times indicated at 10^7^ B cells/ml per condition in a 12-well plate. There were insufficient cells from IκBε−/− mouse 2, following selection, to carry out stimulation of its B cells. Nuclear and cytoplasmic extracts from resting and stimulated cells were analysed for nuclear NF-κB and IκB by immunoblot. Nuclear and cytoplasmic c-Rel were normalised to actin and expressed relative to values for B cells of IκBε+/+ mouse 1 (mouse numbers allocated randomly), as indicated.

Splenic B lymphocytes were obtained from three each of age- and sex-matched WT and IκBε−/− mice. Cells were >93% B220+ by flow cytometry after CD19+ positive selection (not shown). First, we determined levels of c-Rel, p65 and IκB isoforms in nuclear and cytoplasmic extracts of B cells cultured in the presence or absence of stimulatory anti-IgM for up to one hour. Basal levels of nuclear c-Rel relative to nuclear actin were higher in naïve B cells from three IκBε−/− mice compared to those of WT (Figure 7, left side panel 1). Stimulation for 0.5 hour with anti-IgM induced further nuclear translocation of c-Rel in WT and IκBε−/− B cells and this translocation was much stronger in B cells from IκB−/− mice (Figure 7, right side panel 1). After 1 hour's stimulation, nuclear c-Rel in WT B cells approached levels seen for IκBε−/− B cells. Upon re-probe of the same blot, basal nuclear p65 was not detected in WT or IκBε−/− B cells, and p65 was only faintly detectable in WT B cell nuclear extracts after 1 hour's BCR stimulation (Figure 7, panels 2). p65 was strongly detected in one of the IκBε−/− B cell nuclear extracts at this time-point, but the sample appears to be overloaded (see corresponding actin signal in panel 3). Cytosolic expression of p65 was similar in WT and IκBε−/− B cells throughout (Figure 7, panels 5). IκBε was detected in WT but not IκBε−/− B cell cytosols and was degraded following BCR ligation (Figure 7, panels 6). Nuclear expression of c-Rel was enhanced in the absence of IκBε even though IκBα expression was higher in IκBε−/− than in WT B cells (Figure 7, panel 6), as had been the case for T cells. These data strengthen the case for a non-redundant role of IκBε in the regulation of c-Rel in lymphocytes.

### Enhanced survival of naïve IκBε−/− B cells is characterised by increased basal expression of CD40 and higher expression of BAFF-R *ex vivo*

**Figure 8:**
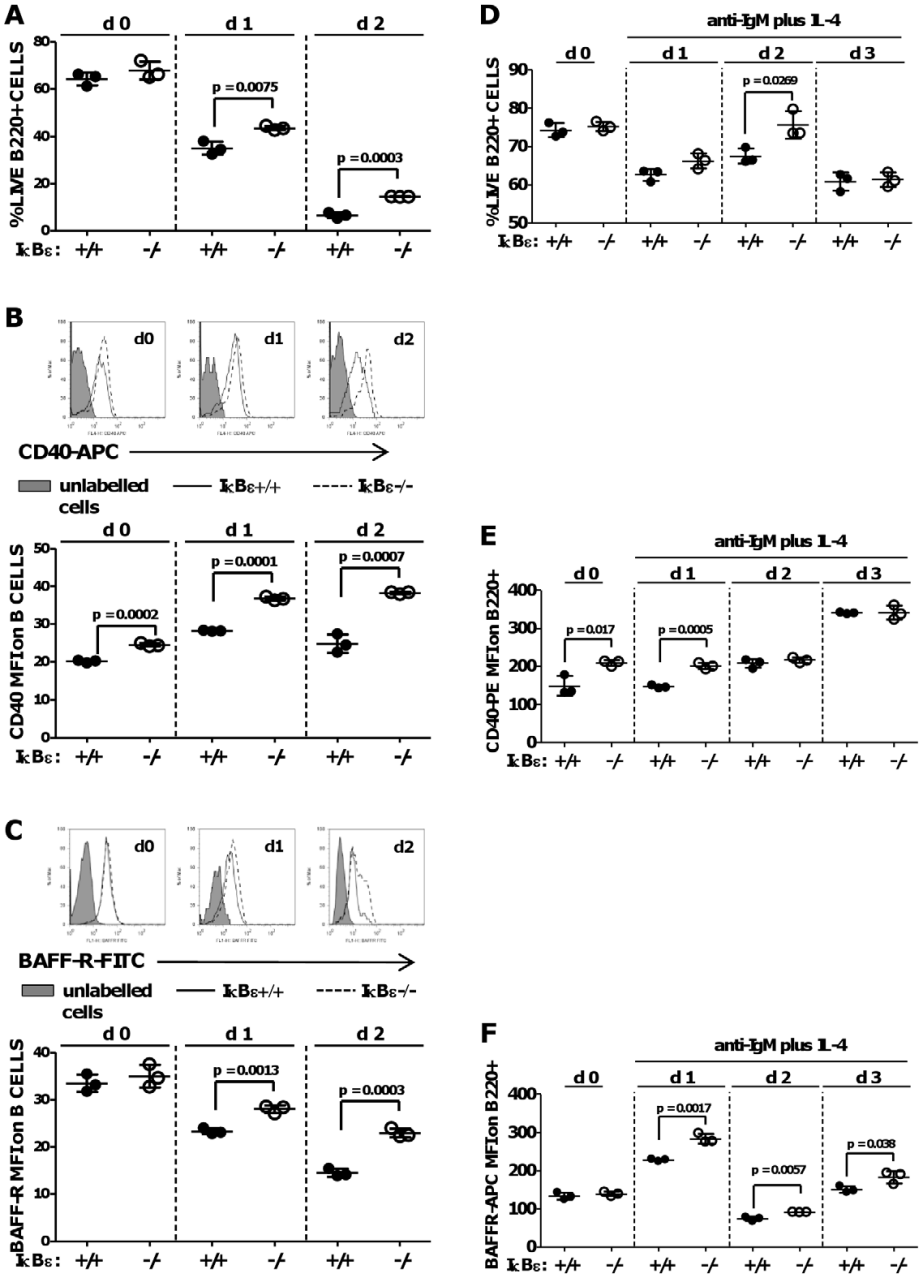
Enhanced survival, increased basal expression of CD40 and increased upregulation of BAFF-R *ex vivo* in IκBε−/− splenic B cells. Splenic B cells were isolated by CD19 positive selection from three each of IκBε+/+ and IκBε−/− mice, plated at 10^6^/ml in 6-well plates without stimulation for 48 hours, and analysed by flow cytometry for (A) viability, as% B220+ cells in the live cell gate (FSc/SSc), (B) median fluorescent intensity (MFI APC) due to CD40 expression on CD40+ live B cells, (C) MFI FITC due to BAFF-R expression on BAFF-R+ live cells. Histograms are for one set of randomly paired IκBε+/+ and IκB−/− B cells, representative of three for each genotype. In a different experiment, splenic B cells were isolated and plated as above, then stimulated with anti-IgM F(ab')_2_ fragment 10 µg/ml and anti-IL-4 50 ng/ml (∼250 u/ml) for 72 hours. Splenic B cells were analysed for (D)% viability (FSc/SSc), (E) CD40 expression (MFI PE), and (F) BAFF-R (MFI APC) by flow cytometry.

To test whether enhanced nuclear translocation of c-Rel affected lymphocyte survival, splenic B cells from 3 WT and 3 IκBε−/−mice were maintained for up to 72 hours either without antigen receptor stimulation (Figure 8 A–C), or in the presence of anti-IgM and IL-4 (Figure 8, D-F). TNF (20 ng/ml) was included in some cultures to test whether it affected expression of IκBε, or behaviour of WT B cells *ex vivo*. Viability of cells was determined by flow cytometry as the percentage of B220+ cells in the live cell gate and in order to explore the mechanism of enhanced survival, expression of c-Rel dependent pro-survival receptors CD40 and BAFF-R on live B220+ cells was determined at the same time.

In the absence of BCR ligation, mean viability of B cells fell rapidly between d0 and d1, and further from d1 to d2. Viability at both d1 and d2 was significantly higher in IκBε−/− B cells, compared to WT cells (Figure 8B). Basal expression of CD40, and its increased expression at d1 and d2 *ex vivo*, were also significantly higher in IκBε−/− B cells (Figure 8B). Basal expression of BAFF-R was similar for WT and IκBε−/− B cells. However, BAFF-R expression decreased with time in culture faster for WT than for IκBε−/− B cells, so that there were significant differences in expression at d1 and d2 *ex vivo* (Figure 8C).

In the case of B cells receiving a pro-survival signal via anti-IgM IL-4, there was a similar loss of viability in both WT and IκBε−/− cells after one day in culture d1 of culture (Figure 8D). Viability appeared to recover between d1 and d2 and, at d2, was significantly greater for IκBε−/− than for WT B cells. Basal expression of CD40 and its expression at d1 of culture in anti-IgM and IL-4 was significantly higher in IκBε−/− B cells than WT B cells (Figure 8E). CD40 expression was increased at d2 and d3 to a similar level in both populations. Basal expression of BAFF-R was similar for WT and IκBε−/− B cells and BAFF-R was strongly induced in both populations after 24 hours in culture, with significantly higher expression on IκBε−/− compared to WT cells B (Figure 8F). The difference persisted and although less marked, it remained statistically significant at d2 and d3 in culture (Figure 8C, D). TNF had no effect on IκBε expression or survival ex vivo of WT B cells, to 72 hours (not shown). These data suggest a non-redundant role for IκBε in the regulation of c-Rel-dependent B lymphocyte survival mechanisms.

## Discussion

While studying mechanisms whereby pre-culture in TNF of mouse T cell hybridoma 11A2 blocked subsequent IL-2 induction, we observed that inhibition of IL-2 was associated with suppressed nuclear translocation of c-Rel as well as increased expression and attenuated inducible degradation of IκBε, but not IκBα or -β. Nuclear expression of c-Fos, Fos B and NFAT2, and transcriptional activation of not only NF-κB, but AP-1 and NFAT/AP-1 reporter promoters, were also attenuated in TNF-treated cells. Deficits in AP-1 and NFAT expression and function are likely to contribute to reduced inducibility of IL-2 in TNF-treated cells both directly and indirectly, since induction of both IL-2 and c-Rel require AP-1 and NFAT2 [26], [30], [50], [51]. Transient reconstitution of c-Rel in TNF-treated cells restored transcriptional activation of a c-Rel/AP-1-dependent reporter to untreated control cell levels but not to those of control cells also over-expressing c-Rel. Therefore c-Rel alone did not fully reconstitute the inhibitory effect of TNF at this promoter. It may be that effects of TNF on AP-1 and NFAT expression and transcriptional activity accounted for this deficit. TNF very likely had pleiotropic effects on 11A2 cells.

The consistent observations that c-Rel translocation was strongly inhibited and that expression of IκBε, but not IκBα or -β, was strongly upregulated in TNF-treated cells led us to investigate the functional relationship of IκBε and c-Rel. Extending our studies to primary lymphoid cells, we found that IκBε appears to have a non-redundant role in regulation of c-Rel subcellular localisation in B lymphocytes and T cell blasts, and a negative regulatory role in B cell survival. We propose that IκBε regulates the latter, at least in part, by inhibiting c-Rel-dependent expression of anti-apoptotic molecules BAFF-R and CD40 in B cells.

Reciprocity of IκBε levels and nuclear translocation of c-Rel held for both TCR- and TNF-R-activated pathways in 11A2 cells at all time-points of stimulation. Basal IκBε expression was increased and remained higher at all time-points of stimulation with PMA and ionomycin, or acute-on-chronic TNF, in TNF pre-treated cells relative to controls. When we examined IκB/NF-κB complexes by co-immunoprecipitation we observed that, whereas c-Rel associated constitutively almost exclusively with IκBβ, newly synthesised c-Rel induced by PMA and ionomycin also associated with IκBα and IκBε. This stimulation-dependent shift in IκB/c-Rel complexes appears analogous to that (from IκBβ to IκBα) documented for cytokine-treated T cells [49]. The amount of newly-synthesised c-Rel associating with IκBα reflected cytosolic levels of c-Rel (Figure 4A, panel 1 and Figure S4 A(ii)). By contrast, its association with IκBε reflected expression levels of IκBε in control and TNF-treated cells (Figure 4A, panel 7 and Figure S4 A(ii)). In addition, more IκBε co-immunoprecipitated with p65/c-Rel in TNF-treated than in control cell lysates of resting and PMA and ionomycin-stimulated cells (Figure 4B, panel 5 and Figure S4B). These were the only TNF-dependent differences in IκB/NF-κB complexes, and seem likely to be a consequence simply of the increased, stable expression of IκBε in TNF-treated cells.

Although its association with IκBε is most marked for newly-synthesised c-Rel, IκBε over-expression also appeared to inhibit nuclear translocation of constitutively-expressed c-Rel. Most c-Rel was associated with IκBβ in resting cells (Figure 4B, panel 4). Yet following TNF-R ligation, despite equivalent degradation of IκBβ (and IκBα) in control and TNF pre-treated cells, very little pre-existing c-Rel reached the nucleus in the latter (Figure 2B panel 2; cf. Figure 2D panels 1–3). One explanation for this may be that IκBε, being so strongly expressed in TNF pre-treated cells, binds and inhibits c-Rel released in the wake of IκBβ degradation.

We found IκBε expression to be upregulated in TNF-treated cells, in all our experiments, at about the same time as another study identified IκBε as a gene directly inducible by TNF [52]). We also noted that IκBε appeared resistant to inducible degradation in TNF-treated cells in many but not all experiments. That there may be mechanisms of its regulation unique to IκBε is suggested not only by its structural difference from the other IκBs [8] but by IκBε-specific interactions with epsilon-regulatory proteins such as PP6 regulatory unit 1(PP6R1) and ankyrin repeat subunit (ARS)-A of PP6 which have recently been described. Knock-down of either the PP6R1scaffolding unit or of ARS-A (thought to mediate phosphatase substrate specificity) led to enhanced TNF-induced degradation of IκBε [18], [19], [20].

IκBε is expressed in T and B lymphocytes [9], [47] and it has been shown that IκBε, like IκBβ, prefers to bind c-Rel and p65 homo- or heterodimers. This is in contrast to IκBα which preferentially binds p65/p50 [8], [10]. IκBε is expressed at high levels in splenic B cells, in which it has been suggested to have a role in regulating a subset of genes activated by p65 homodimers, since p65 homodimer-driven reporter gene expression increased as IκBε was down-regulated during B cell maturation [9]. The same authors had previously described constitutive nuclear c-Rel expression in IgG+ but not IgM+ murine B cell lines, with 10-fold lower expression of IκBε in the former.[53]. We found that culture of WT murine T cell blasts (8 days) or splenic B cells (3 days) in TNF did not upregulate IκBε above its already-high expression levels in the absence of TNF. It has further been reported that c-Rel is constitutively nuclear in mature but not naïve B cells [23]. We extended our investigation of specific regulation of c-Rel by IκBε, and its functional outcomes, to studies in lymphoid cells of WT and IκBε−/− mice.

We argued that if IκBε had a unique role in regulation of c-Rel nuclear translocation, as our *in vitro* experiments suggested, c-Rel would accumulate in the nucleus of lymphocytes lacking IκBε, in the presence of normal levels of expression of IκBα and -β. We looked first in T cell blasts and found that c-Rel was present in the nucleus of resting IκBε−/− and IκBε+/− T cells in the absence of TCR signal (Figure 5A, B,C) and that this occurred despite increased expression of IκBα and, to a lesser extent, IκBβ. TCR-induced nuclear translocation of c-Rel was seen for WT T cell blasts, with less marked further nuclear accumulation of c-Rel in IκBε−/− T cells, suggesting that nuclear c-Rel was already closer to its maximum in the latter. Nuclear c-Rel was also present at higher levels in naïve splenic IκBε−/− B cells, relative to WT, and its early BCR-induced translocation was much more marked in IκBε−/− B cells than WT (Figure 7). Again, accumulation of nuclear c-Rel occurred in IκBε−/− B cells in the presence of higher IκBα expression relative to WT cells. c-Fos and NFAT2 were not detected in nuclei of resting or stimulated B cells or T cell blasts.

When we studied functional outcomes of enhanced c-Rel nuclear translocation, we were surprised that IκBε−/− T cells (naïve or blast cells) did not make more IL-2 than WT cells when stimulated via TCR *ex vivo*, and showed only a modest increase in potential for IL-2 synthesis when stimulated with PMA and ionomycin (Figure 5E). However, these findings were consistent with a study which showed that inhibition of IL-2 expression in c-Rel−/− relative to that in wild-type T cells was not detectable before 4 hours of TCR stimulation, that is, until the time when de-novo synthesised c-Rel would accumulate in the nucleus [26]. The authors also showed that basal and inducible transcription of IL-2 in T cells over-expressing c-Rel was between 2 and 3-fold greater than in control cells but that this difference was lost at four hours' stimulation when the gene was very strongly upregulated. By way of comparison, basal and TCR-induced CD40 expression in c-Rel−/− T cells were inhibited at all time-points, relative to wild-type, and c-Rel over-expression led to basal and inducible transcription of CD40 that was between 15 and 40-fold greater than that of controls throughout. We would argue that basal nuclear expression of endogenous c-Rel in T lymphocytes is actually unlikely to lead to enhanced IL-2 expression as a result of the kinetics of regulation of this gene: TCR-induced activation of IL-2 requires *de novo* synthesis of c-Rel, AP-1 and NFAT proteins and their coordinate binding at pIL-2. Peak expression of these factors coincides with maximum degradation of IκBε translocation in wild type cells. Thus at the time (from about 2 hours post-stimulation) when TCR-induced transcription of IL-2 would be expected to begin in earnest, there would be effectively no difference in IκBε between IκBε−/− and WT cells. However, under circumstances in which its expression is increased above basal levels – in response to TNF, for instance, should this occur *in vivo* – a role for IκBε in negative regulation of IL-2 gene induction might become apparent.

Further observations led us to explore c-Rel responsive genes besides IL-2 in IκBε−/− lymphocytes. Firstly, naïve splenocytes and LNCs of IκBε−/− mice showed enhanced sensitivity to low concentrations of anti-CD3, by thymidine incorporation assay, and we consistently saw increased basal thymidine incorporation in IκBε−/− splenocyte, LNC and T cell blast cultures (Figures 5F and 6A). ^3^H-thymidine uptake is usually taken as a correlate of IL-2-dependent proliferation in T cell cultures, but it depends on the number of live cells in culture at the time of its addition – typically 48 hours into culture – and so it also depends on cell survival *ex vivo* [54]. Basal ^3^H-thymidine incorporation showed a reciprocal relationship with IκBε gene dose which was not due to IL-2. This might be explained either by increased basal proliferation or enhanced survival *ex vivo* of IκBε−/− cells. That is, if the viability of IκBε−/− cells exceeded that of WT cells, there would be more of the former able to take up thymidine 48 hours into culture. Secondly, LNs from IκBε−/− mice, which often looked slightly larger than those from WT mice, contained significantly increased LNC numbers, with a trend to a higher proportion of B cells (Figure 6, B–D). Thirdly, B cells from IκBε−/− but not WT mice survived for up to 8 days in conditions favouring T cell expansion and survival.

From the data, we felt we were looking at a IκBε−/− lymphoid cell phenotype characterised chiefly by enhanced survival mechanisms most clearly manifest in B cells. This interpretation accords with previous adoptive transfer experiments which showed a significant increase in splenic B cells of IκBε−/− chimaeras as compared to wild-type or IκBα mutant ones [55]. Having established that naïve IκBε−/− splenic B cells showed increased basal nuclear expression and BCR-induced nuclear translocation of c-Rel compared to WT, we found that survival of these cells was significantly enhanced for up to 48 hours *ex vivo* in the presence or absence of BCR ligation (Figure 8A and 8D). We demonstrated significantly greater basal expression of CD40 in naïve IκBε−/− splenic B cells relative to WT which persisted for 24 or 48 hours in cells cultured in the presence or absence of anti-IgM and IL-4 respectively (Figure 8B and 8E). Finally, we observed a statistically significant persistence of BAFF-R expression in unstimulated IκBε−/− B cells relative to WT for up to 48 hours culture *ex vivo* (Figure 8C), and enhanced BCR-induced upregulation of BAFF-R in IκBε−/− compared to WT B cells, which was most marked at 24 hours, persisted for a further 48 hours (Figure 8D) and was also statistically very significant. The higher basal and inducible expression levels of these c-Rel-dependent pro-survival molecules is a likely mechanism for the enhanced survival of IκBε−/− B cells *ex vivo*. CD40- and BAFF-R-dependent survival of B cells *ex vivo* could in turn account for the increased basal thymidine uptake *ex vivo* of IκBε−/− splenocytes, LNCs and unstimulated T cell cultures, relative to WT, both through B cell survival and the ability of those B cells to deliver co-stimulation to T cells. Enhanced B cell co-stimulation might also explain the greater sensitivity of these cultures to low concentrations of anti-CD3.

Doerre and colleagues proposed an important role for IκBε in differential regulation of genes responsive to p65 homodimers in stimulated B cells [9]. A recent study describes IκBε as having a specific role in regulating random variation of NF-κB activity from cell to cell such as to permit cellular heterogeneity and differentiation [56]. We propose that IκBε is a non-redundant regulator of c-Rel in both T and B cells and that IκBε deficiency or down-regulation leads to enhanced expression of c-Rel-dependent genes that permit survival of, at least, B lymphocytes. Given such effects on lymphoid cell survival, and the fact that c-Rel is a proto-oncogene, we wondered whether IκBε−/− mice were predisposed to lymphatic tumours. However, no overt tumour was detected in lymphoid tissues of male or female mice aged up to 14 months. With hindsight, this is not surprising, since there are no reports of transformation mediated solely by over-expression of c-Rel in mice [23]. However, studies have implicated gene variants in the IκBε/c-Rel pathway in tumorigenesis: mutations in CD40, c-Rel and IκBε are associated with Hodgkin's Disease, a malignant transformation of germinal centre B cells. In terms of other disease risks, rheumatoid arthritis-associated single nucleotide polymorphisms have been described in PKCθ, which lies upstream of TCR-induced IκB degradation, in c-Rel, and in c-Rel target genes CD40 and TNFAIP3 [57], [58], [59]; and IκBε itself is upregulated at the mRNA level by TNF, whose over-expression is a feature of rheumatoid arthritis and other inflammatory diseases [52].

Our data suggest strongly that dysregulated expression of IκBε will alter c-Rel-dependent survival and function of lymphocytes, its under-expression or impaired function leading to enhanced lymphocyte survival, and its over-expression or resistance to degradation leading to impaired lymphocyte survival or function. Identification of perturbations in this pathway in the context of human disease is worthy of further study.

## Supporting Information

Figure S1 **PMA plus ionomycin-induced IL-2 mRNA is stable in TNF-treated cells.** Control and TNF-treated 11A2 cells were stimulated for four hours with P+I_low_ prior to addition of actinomycin D 10 µg/ml. Cells were harvested at the times indicated and RNA extracted and analysed for IL-2 mRNA by ribonuclease protection assay. (A) Phosphorimage of protected RNA species; (B) IL-2 mRNA normalised to L32 mRNA and expressed as% peak levels (at time of addition of actinomycin D) for control and TNF-treated cells. Mean +/− SD, 3 experiments.
(PDF)

Figure S2 **Constitutive nuclear NFAT1 in 11A2 cells. Dephosphorylation and nuclear translocation of NFAT1 in control and TNF-pre-treated cells.** 11A2 cells were cultured with or without TNF 2.5 ng/ml for 8 days before restimulation with PMA 10 ng/ml and ionomycin 50 ng/ml (P+I)_low_ for the times indicated. Nuclear and cytoplasmic extracts were prepared and equivalent amounts of proteins assayed for the presence of NFAT1 by immunoblot. This filter was subsequently re-probed for NFAT2 and loading controls (see Figure 1D). Representative of three similar experiments.
(PDF)

Figure S3 **Attenuation of DNA-binding for AP-1 proteins and NFAT2 in TNF-treated cells.** Control and TNF-treated cells were stimulated for 4 hours with P+I_high_. Nuclear extracts were incubated with ^32^P-labelled (A)(B) AP-1 or (C) NFAT/AP-1 oligonucleotide, with or without supershifting antibodies for (A) fos proteins, (B) jun proteins or (C) NFAT2. Protein-bound oligonucleotide was visualised by phosphorimaging after gel electrophoresis.
(PDF)

Figure S4 **NF-κB and IκB in input cytosol and post-immunoprecipitate supernatants.** Control and TNF-treated 11A2 cells were stimulated with (P+I)_low_ for the indicated times. Nuclear and cytosolic extracts were prepared and either (A) IκBs (α, β, ε), or (B and C) p65 immunoprecipitated from 100 µg cytosolic protein. (A) (i) IκB and (ii) NF-κB in input and post-IP supernatants following immunoprecipitation with anti-IκB, 4 hour time-point of PMA and ionomycin stimulation; (B) cytosolic NF-κB and IκB in input and (C) input at t = 0 h, and post-IP supernatants following immunoprecipitation with anti-c-Rel or anti-p65, at t = 0 and 2 h PMA + ionomycin stimulation.
(PDF)

Table S1 **Basal IκB and nuclear NF-κB expression in IκBε sufficient, heterozygous and deficient murine T cell blasts.** Splenocytes and LNCs from IκBε+/+, IκBε+/− or IκBε−/− mice, were cultured with anti-CD3 for 48 hours, washed, then stimulated with IL-2 at day 2 and day 5. T cell blasts were harvested on day 7. Nuclear and cytoplasmic extracts were analysed for NF-κB and IκB by immunoblot. Following scanning densitometry and quantitation, band volumes for each protein were normalised against the corresponding value for actin. Mean +/− SD, n = 3 experiments.
(DOC)
