# Supplementary material for: Inhibitor of Kappa B Epsilon (IκBε) Is a Non-Redundant Regulator of c-Rel-Dependent Gene Expression in Murine T and B Cells
Source: PLoS One. 2011 Sep 6;6(9):e24504. doi: 10.1371/journal.pone.0024504 (PMC3167847; doi:10.1371/journal.pone.0024504)
Supplement: Figure S1 — PMA plus ionomycin-induced IL-2 mRNA is stable in TNF-treated cells. Control and TNF-treated 11A2 cells were stimulated for four hours with P+Ilow prior to addition of actinomycin D 10 µg/ml. Cells were harvested at the times indicated and RNA extracted and analysed for IL-2 mRNA by ribonuclease protection assay. (A) Phosphorimage of protected RNA species; (B) IL-2 mRNA normalised to L32 mRNA and expressed as% peak levels (at time of addition of actinomycin D) for control and TNF-treated cells. Mean +/− SD, 3 experiments. (PDF) [file pone.0024504.s001.pdf]

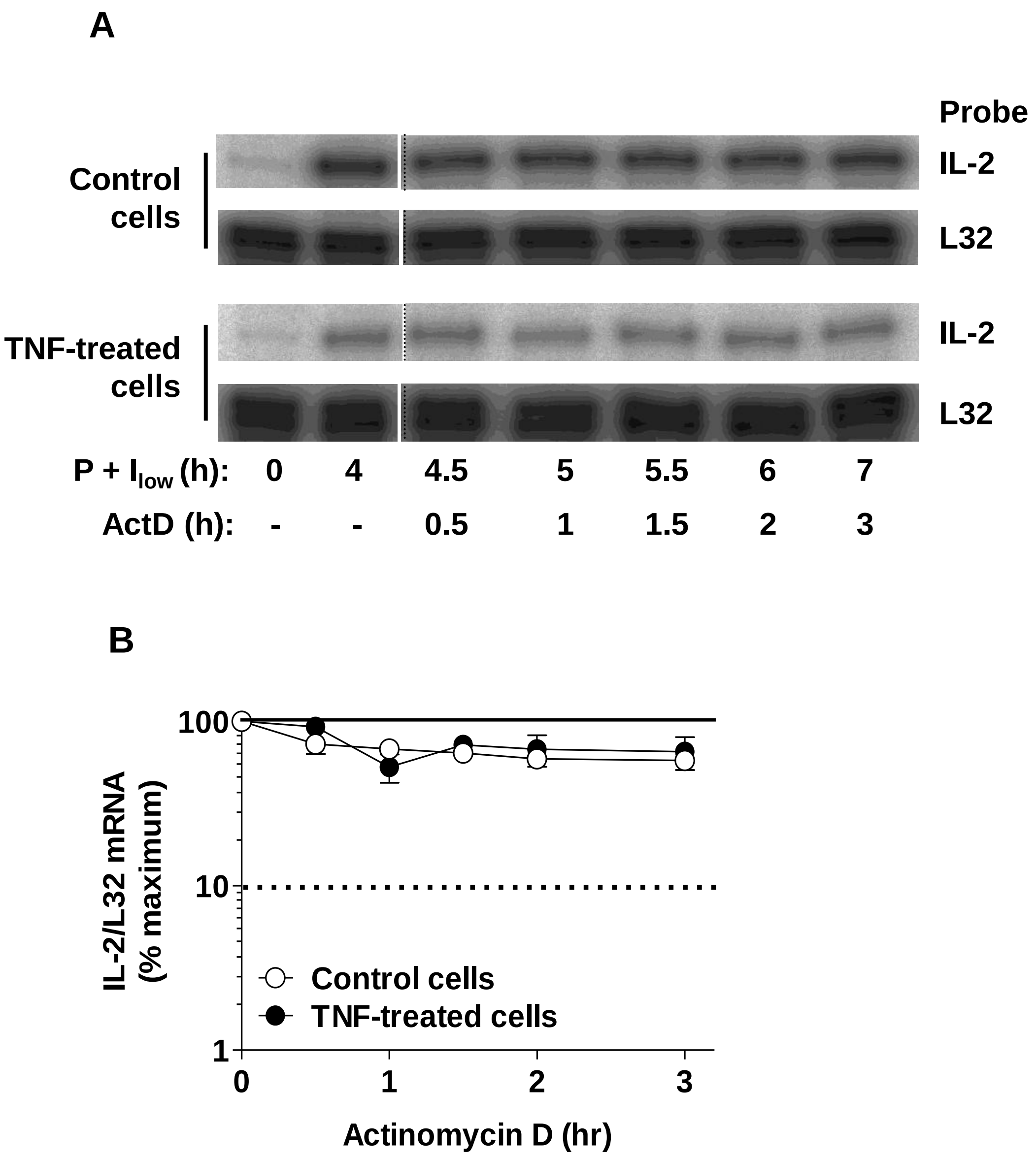


**Figure S1: PMA plus ionomycin-induced IL-2 mRNA is stable in TNF-treated cells.** Control and TNF-treated 11A2 cells were stimulated for four hours with P+$I_{low}$ prior to addition of actinomycin D 10 μg/ml. Cells were harvested at the times indicated and RNA extracted and analysed for IL-2 mRNA by ribonuclease protection assay. (A) Phosphorimage of protected RNA species; (B) IL-2 mRNA normalised to L32 mRNA and expressed as % peak levels (at time of addition of actinomycin D) for control and TNF-treated cells. Mean +/- SD, 3 experiments.
