# Supplementary material for: Inhibitor of Kappa B Epsilon (IκBε) Is a Non-Redundant Regulator of c-Rel-Dependent Gene Expression in Murine T and B Cells
Source: PLoS One. 2011 Sep 6;6(9):e24504. doi: 10.1371/journal.pone.0024504 (PMC3167847; doi:10.1371/journal.pone.0024504)
Supplement: Figure S2 — Constitutive nuclear NFAT1 in 11A2 cells. Dephosphorylation and nuclear translocation of NFAT1 in control and TNF-pre-treated cells. 11A2 cells were cultured with or without TNF 2.5 ng/ml for 8 days before restimulation with PMA 10 ng/ml and ionomycin 50 ng/ml (P+I)low for the times indicated. Nuclear and cytoplasmic extracts were prepared and equivalent amounts of proteins assayed for the presence of NFAT1 by immunoblot. This filter was subsequently re-probed for NFAT2 and loading controls (see Figure 1D). Representative of three similar experiments. (PDF) [file pone.0024504.s002.pdf]

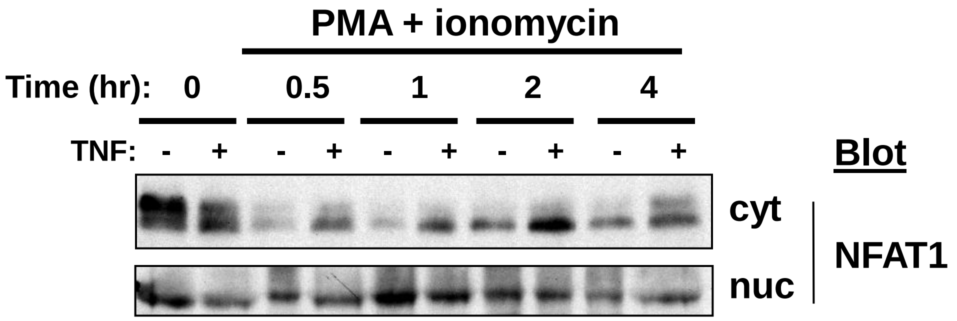

Figure S2: Constitutive nuclear NFAT1 in 11A2 cells. Dephosphorylation and nuclear translocation of NFAT1 in control and TNF-pre-treated cells 11A2 cells were cultured with or without TNF 2.5 ng/ml for 8 days before restimulation with PMA 10 ng/ml and ionomycin 50 ng/ml $(P+I)_{low}$ for the times indicated. Nuclear and cytoplasmic extracts were prepared and equivalent amounts of proteins assayed for the presence of NFAT1 by immunoblot. This filter was subsequently re-probed for NFAT2 and loading controls (see Fig. 1D) Representative of three similar experiments.
