# Supplementary material for: Inhibitor of Kappa B Epsilon (IκBε) Is a Non-Redundant Regulator of c-Rel-Dependent Gene Expression in Murine T and B Cells
Source: PLoS One. 2011 Sep 6;6(9):e24504. doi: 10.1371/journal.pone.0024504 (PMC3167847; doi:10.1371/journal.pone.0024504)
Supplement: Figure S3 — Attenuation of DNA-binding for AP-1 proteins and NFAT2 in TNF-treated cells. Control and TNF-treated cells were stimulated for 4 hours with P+Ihigh. Nuclear extracts were incubated with 32P-labelled (A)(B) AP-1 or (C) NFAT/AP-1 oligonucleotide, with or without supershifting antibodies for (A) fos proteins, (B) jun proteins or (C) NFAT2. Protein-bound oligonucleotide was visualised by phosphorimaging after gel electrophoresis. (PDF) [file pone.0024504.s003.pdf]

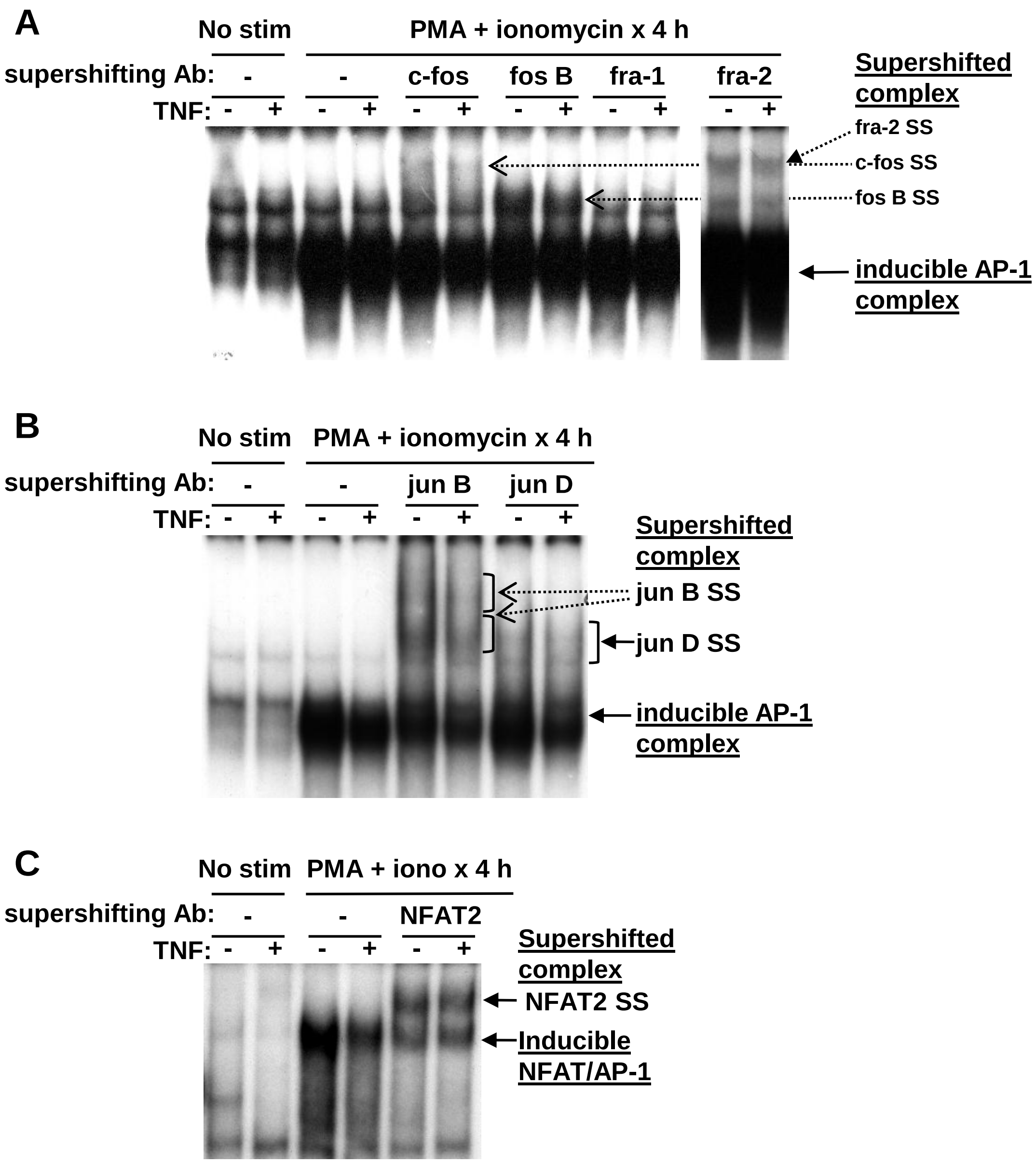

Figure S3: Attenuation of DNA-binding for AP-1 proteins and NFAT2 in TNF-treated cells. Control and TNF-treated cells were stimulated for 4 hours with $P+I_{high}$. Nuclear extracts were incubated with ^{32}P-labelled (A)(B) AP-1 or (C) NFAT/AP-1 oligonucleotide, with or without supershifting antibodies for (A) fos proteins, (B) jun proteins or (C) NFAT2. Protein-bound oligonucleotide was visualised by phosphorimaging after gel electrophoresis.
