# Supplementary material for: Inhibitor of Kappa B Epsilon (IκBε) Is a Non-Redundant Regulator of c-Rel-Dependent Gene Expression in Murine T and B Cells
Source: PLoS One. 2011 Sep 6;6(9):e24504. doi: 10.1371/journal.pone.0024504 (PMC3167847; doi:10.1371/journal.pone.0024504)
Supplement: Figure S4 — NF-κB and IκB in input cytosol and post-immunoprecipitate supernatants. Control and TNF-treated 11A2 cells were stimulated with (P+I)low for the indicated times. Nuclear and cytosolic extracts were prepared and either (A) IκBs (α, β, ε), or (B and C) p65 immunoprecipitated from 100 µg cytosolic protein. (A) (i) IκB and (ii) NF-κB in input and post-IP supernatants following immunoprecipitation with anti-IκB, 4 hour time-point of PMA and ionomycin stimulation; (B) cytosolic NF-κB and IκB in input and (C) input at t = 0 h, and post-IP supernatants following immunoprecipitation with anti-c-Rel or anti-p65, at t = 0 and 2 h PMA + ionomycin stimulation. (PDF) [file pone.0024504.s004.pdf]

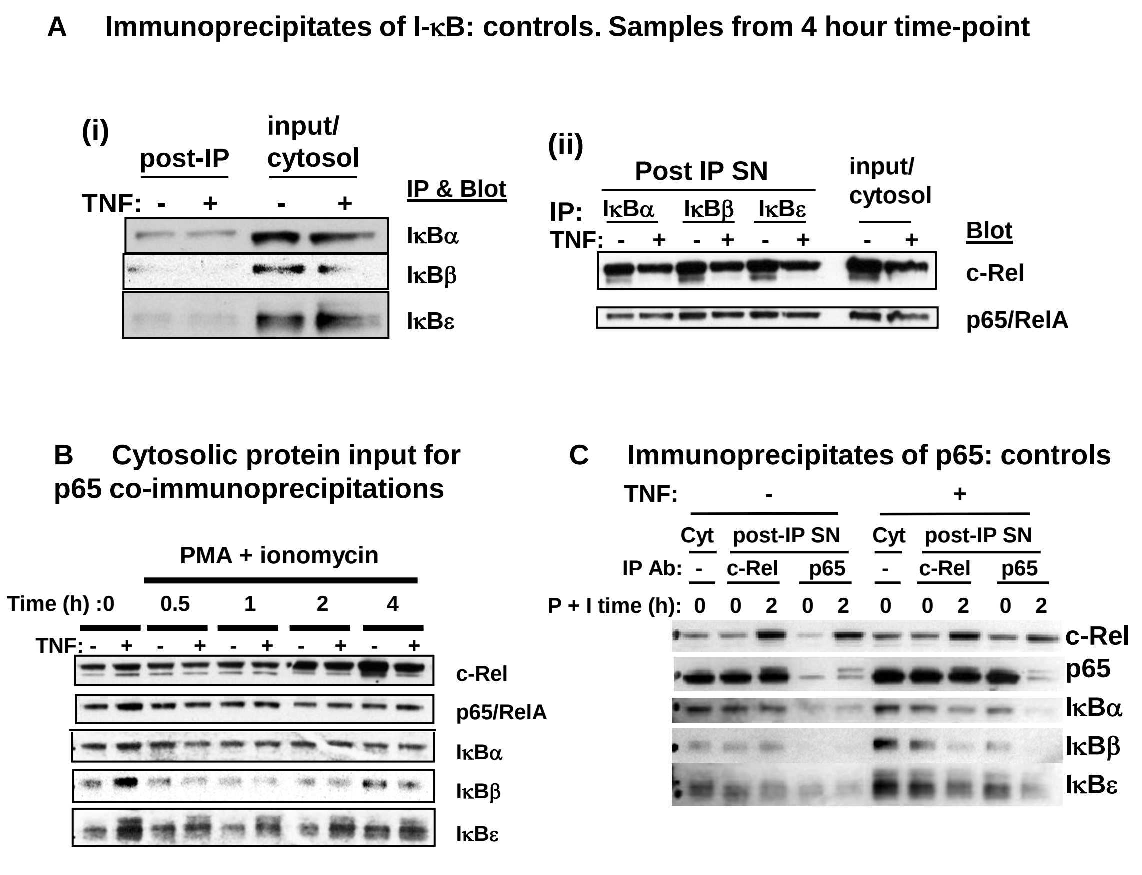

Figure S4: NF-κB and IκB in input cytosol and post-immunoprecipitate supernatants. Control and TNF-treated 11A2 cells were stimulated with $(P+I)_{low}$ for the indicated times. Nuclear and cytosolic extracts were prepared and either (A) IκBs (α, β, ε), or (B and C) p65 immunoprecipitated from 100 μg cytosolic protein. (A) (i) IκB and (ii) NF-κB in input and post-IP supernatants following immunoprecipitation with anti-IκB, 4 hour time-point of PMA and ionomycin stimulation; (B) cytosolic NF-κB and IκB in input and (C) input at t = 0h, and post-IP supernatants following immunoprecipitation with anti-c-Rel or anti-p65, at t = 0 and 2h PMA + ionomycin stimulation.
