# Supplementary material for: Inhibitor of Kappa B Epsilon (IκBε) Is a Non-Redundant Regulator of c-Rel-Dependent Gene Expression in Murine T and B Cells
Source: PLoS One. 2011 Sep 6;6(9):e24504. doi: 10.1371/journal.pone.0024504 (PMC3167847; doi:10.1371/journal.pone.0024504)
Supplement: Table S1 — Basal IκB and nuclear NF-κB expression in IκBε sufficient, heterozygous and deficient murine T cell blasts. Splenocytes and LNCs from IκBε+/+, IκBε+/− or IκBε−/− mice, were cultured with anti-CD3 for 48 hours, washed, then stimulated with IL-2 at day 2 and day 5. T cell blasts were harvested on day 7. Nuclear and cytoplasmic extracts were analysed for NF-κB and IκB by immunoblot. Following scanning densitometry and quantitation, band volumes for each protein were normalised against the corresponding value for actin. Mean +/− SD, n = 3 experiments. (DOC) [file pone.0024504.s005.doc]

**Table S1: Basal IκB and nuclear NF-κB expression in IκBε sufficient, heterozygous and deficient murine T cell blasts.**

|  | **Normalised band volume (arbitrary units)** | | |
| --- | --- | --- | --- |
|  | **I-κBε genotype** | | |
| **Cytosolic I-κB** | **+/+** | **+/-** | **-/-** |
| I-κBα | 0.49 +/- 0.27 | 0.56 +/- 0.17 | 0.73 +/- 0.22 |
| I-κBβ | 0.54 +/- 0.13 | 0.55 +/- 0.17 | 0.62 +/- 0.22 |
| I-κBε | 1.05 +/- 0.33 | 0.79 +/- 0.09 | 0 |
| **Nuclear NF-κB** |  |  |  |
| p65 | 0.015 +/- 0.03 | 0.05 +/- 0.03 | 0.06 +/- 0.03 |
| c-Rel | 0.08 +/- 0.02 | 0.17 +/- 0.04 | 0.29 +/- 0.01 |
| p50 | 0 | 0 | 0 |

Splenocytes and LNCs from IκBε+/+, IκBε+/- or IκBε-/- mice, were cultured with anti-CD3 for 48 hours, washed, then stimulated with IL-2 at day 2 and day 5. T cell blasts were harvested on day 7. Nuclear and cytoplasmic extracts were analysed for NF-κB and IκB by immunoblot. Following scanning densitometry and quantitation, band volumes for each protein were normalised against the corresponding value for actin. Mean +/- SD, n = 3 experiments.
